# Measuring Electrode Kinetics Under Spatial Confinement. Application of Pulse Voltammetry to Different Mass Transport Modes Using Butler–Volmer and Marcus–Hush–Chidsey Frameworks

**DOI:** 10.1021/acsmeasuresciau.6c00096

**Published:** 2026-05-26

**Authors:** José V. Hernández-Tovar, Antonio J. Martínez-García, Francisco Martínez-Ortiz, Eduardo Laborda, Manuela López-Tenés, Joaquín González

**Affiliations:** Departamento de Química Física, Facultad de Química, Regional Campus of International Excellence “Campus Mare Nostrum”, 16751 Universidad de Murcia, Murcia 30100, Spain

**Keywords:** finite diffusion, thin-layer, voltammetry, normal pulse voltammetry, chronoamperometry, Butler−Volmer, Marcus−Hush−Chidsey

## Abstract

Spatial confinement arises in many electrochemical phenomena, such as the electrochemical characterization of ultrathin membranes; insertion of ionic species into porous matrices; charge transfer processes in porous electrodes or through porous matrices; or redox cycling devices with bipotentiostatic control. In all these situations the mass transport occurs in solution layers whose thickness is comparable to or smaller than that of the diffusion layer. Two mass transport modalities can be considered in this context of finite diffusion: one leading to the partial or complete depletion of the electroactive species and the other related to the faradaic regeneration of the redox species. Of special interest is the relationship between spatial confinement and kinetic influences on overall electrochemical responses. In the specific case of redox kinetics, there is a lack of theoretical frameworks developed specifically for these new experimental scenarios, in order to provide simple and accurate tools that enable us to characterize the kinetics at play. In fact, methodologies developed for semi-infinite diffusion (as, for example, the Nicholson method) are still being applied, even though they are clearly unsuitable in this case. Normal Pulse Voltammetry (NPV) is proposed as a simple and accurate technique to characterize electrochemical processes with slow kinetics. Unusual features arise in the current–potential responses due to the overall kinetic dependence, which combines the effects of mass transport and redox kinetics. This allows all the key parameters of the responses to be determined with great accuracy. The well-known Butler–Volmer (BV) and Marcus–Hush–Chidsey (MHC) formalisms have been considered. This technique was applied to analyze the quasi-reversible oxidation of 4-carboxy-2,2,6,6-tetramethylpiperidine-1-oxyl (4-carboxy-TEMPO) radicals in aqueous solutions using a boron-doped diamond electrode. MHC formalism was found to be more coherent when describing the electrochemical responses for both mass transport modes.

## Graphical abstract

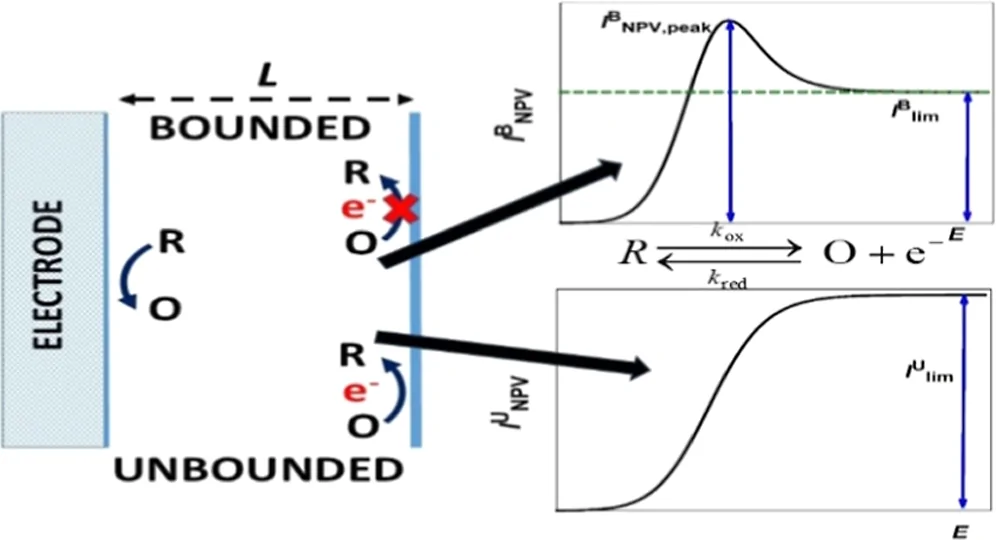

## Introduction

The electrochemical responses of electroactive species in solution are defined by the interplay between interfacial kinetics and mass transport.^1–6^ In the absence of convection and migration, this combined influence is usually addressed by assuming a “semi-infinite” diffusion (SID) field. This means that the electrochemical process takes place at a solution layer much wider than the region adjacent to the electrode that is disturbed by an heterogeneous charge transfer process (i.e., the diffusion layer, for which the concentration of the species undergoing oxidation or reduction differs from their bulk values).^1–3,7^ Over the last 80 years, a sound theoretical knowledge has been developed and successfully applied for this situation, typical of laboratory electrochemical cells.^1–3,8^

Beyond this well-established configuration, there is a growing number of practical situations of interest for which the mass transport component of the response is a spatially restricted diffusion field moving from microscale to nanoscale. Under these conditions, called as spatial confinement, the mass transport is distorted, leading to the appearance of new features in the electrochemical responses of the systems under study. For example, confinement could lead to an increase of the local concentration and effective kinetics of the redox probes, together with the possibility of enhancement or suppression of coexisting chemical reactions, giving rise to new reaction pathways in these environments.^9–11^

Spatial confinement arises in many electrochemical phenomena and devices, such as the electrochemical characterization of thin and ultrathin membranes;^12,13^ the insertion of ionic species into porous matrices;^14–16^ charge transfer processes in porous electrodes or through porous matrices;^17–19^ and, of course, combinations of the above categories, which are typical of multiphasic devices.^20–24^ Additionally, redox cycling devices with bipotentiostatic control typically operate under spatial confinement conditions to enhance the sensitivity of their current or charge responses through adequate device design.^25–28^ All these situations imply a finite diffusion (FD) field; that is to say, the mass transport phenomena occur in solution layers whose thickness is comparable to or smaller than that of the diffusion layer. Two mass transport modalities can be considered in this context corresponding to different physicochemical conditions: one leading to the partial or complete depletion of the electroactive species and the other related to the faradaic regeneration of the redox species, depending on the architecture of the electrochemical setup. These devices are referred to as Thin-Layer (TL) cells in the literature, but as previously stated, TL behavior is a special case of the more general FD problem.^29,30^ Note that all the above situations correspond to biphasic systems. There exist also the so-called “Thin-film” systems that involve three phases, an electrode, an insoluble film and an electrolyte solution, which will not be considered here.^31,32^

A recurrent methodological problem that arises when analyzing the above-listed situations, in which FD is the operating mass transport, is that the established criteria for determining rate constants, diffusion coefficients and thermodynamic parameters have been obtained under SID conditions. Therefore, they are clearly not applicable to these new environments.^29,30,33,34^ Nevertheless, it is not unusual to see the Randles–Sevčik equation applied to the analysis of Cyclic Voltammetry responses, Cottrell equation applied to the analysis of current–time responses, or Nicholson’s method applied to the determination of rate constants.^35–38^ These three well-known expressions are only rigorously valid for SID conditions. Despite this, they have been used to analyze electrochemical responses obtained under spatial confinement conditions. Therefore, new approaches are needed for FD problems.

The first attempt to address the aforementioned issue and gain accurate physicochemical insights into electrochemical processes under spatial confinement involves adapting the frameworks that were previously developed in the 1960s–1980s to describe the response of electrochemical processes under TL conditions, and applying them to these new situations.^29,30,39–44^ While these previous results were based on linear finite diffusion, they can be considered an adequate approximation of practical experimental situations when the diffusion coefficients of the electroactive species are small.

Theoretical analyses conducted under FD conditions were mostly restricted to fast electrochemical reactions.^29,30,43,44^ Nevertheless, the relationship between spatial confinement and kinetic influences on overall electrochemical responses is of particular interest. In the specific case of redox kinetics, several previous approaches to this problem can be found in refs 45–48 , but none of these approaches have developed a general method for quantifying the influence of mass transport under both depletion (bounded diffusion) and faradaic regeneration (unbounded diffusion) of the electroactive species. These references address the electrochemical response of slow kinetic processes in cyclic voltammetry, square wave voltammetry and chronoamperometry. Furthermore, previous efforts have focused on the Butler–Volmer (BV)^1–3^ formalism and not considered the more realistic Marcus–Hush–Chidsey (MHC) theory.^49–51^ When describing the heterogeneous electron transfer kinetics between molecules and electrodes, the former model is broadly used, although it provides a simplified and phenomenological picture and it is not unusual to observe deviations from its predictions. A more accurate formalism as MHC seems to be more adequate for the description of the electrochemical behavior of these processes.^50,52,53^ Despite a higher complexity compared to BV, MHC formalism allows to get more detailed insights into the redox process kinetics, and it provides a more molecular-rooted description of the governing parameters of the process.

As a simple and accurate alternative electrochemical method to those mentioned above, we propose the use of the Normal Pulse Voltammetry (NPV) technique, a single-pulse technique available in commercial electrochemical equipment packages, to characterize electrochemical processes with slow kinetics. NPV involves applying a series of constant potentials of the same duration to the electrode, with the initial conditions being restored between each potential application.^1,3^ Consequently, the response to each potential in the sequence can be considered independent of the others. In the case of spatial confinement, the multipulse modality of this technique can be used if a resting potential is applied for a relatively short period of time between every two potentials in the sequence, thereby regenerating the electroactive species in a very effective way. Furthermore, sampling the current over extended periods renders ohmic distortions in the NPV responses negligible.^3^

We have obtained a general expression for the current–potential-time response when a constant potential is applied to the two mass transport modalities indicated above (bounded and unbounded conditions). First, we have analyzed the chronoamperometric current–time responses for charge transfer processes exhibiting slow redox kinetics. We have then applied the general solution to quantify the NPV responses of quasi-reversible or fully irreversible processes. Unusual features arise in the current–potential responses due to the overall kinetic dependence, which combines the effects of FD mass transport and redox kinetics. This allows all the key parameters of the responses to be determined with great accuracy. Both the BV and MHC formalisms were considered and compared. It is important to note that, since mass transport and kinetic influences are coupled, the two diffusion modalities considered here exhibit opposite kinetic behavior at the TL limit. Consequently, transitioning from SID to FD means that quasi-reversible charge transfer processes appear faster under depletion and slower under regeneration.

The NPV technique was applied to analyze the quasi-reversible oxidation of 4-carboxy-2,2,6,6-tetramethylpiperidine-1-oxyl (4-carboxy-TEMPO) radicals in aqueous solutions using a boron-doped diamond electrode under both bounded and unbounded conditions. The theoretical framework presented here was used to obtain all the kinetic parameters using the BV and MHC formalisms.

### Current-Potential-time Responses under Bounded and Unbounded FD Fields

Let us consider the following heterogeneous slow charge transfer process, taking place at the interface between an electrode and an electrolytic solution due to the application of a constant potential *E*

I $$R\overset{k_{ox}}{\underset{k_{red}}{⇄}}O+e^{-}$$

with R and O being reduced and oxidized species at initial concentrations $c_{R}^{*}$ and $c_{O}^{*}$ , respectively. *k*_ox_ and *k*_red_ are the potential dependent rate constants for the oxidation and reduction processes, respectively. The kinetic formalism that describes the explicit potential dependence of both rate constants will be the broadly used Butler–Volmer one, BV,^1–3,54^ or the molecular-rooted Marcus–Hush–Chidsey, MHC.^2,3,50^ For a more detailed discussion see the expressions of the rate constant given in Supporting Information (see eqs S1–S5 in Section S1).

Here, it will be considered that process (I) is taking place at a narrow solution region of thickness *L* between the electrode surface and a “wall” located at a distance *x* = *L*, which is of the order or smaller than the diffusion layer generated by the heterogeneous charge transfer process. This is a general situation which covers the Semi-Infinite Diffusion limit (SID, in which *L* tends to infinite), and the so-called Thin Layer limit (TL, where *L* tends to zero).^29,30^ The mass transport will be assumed as linear inside this region.

Two mass transport modes will be considered depending on the electroactive or electro-inactive character of the wall located at *x* = *L*. To clearly describe both modes, a schematic picture of the electrochemical cell is given in Scheme 1. The “bounded” diffusion (left figure in Scheme 1) considers an electroinactive wall, such that both species R and O have null flux at this point and the electrochemical conversion of species R takes place only at the inner space of the diffusive region, excluding any possibility of matter exchange (no mass renovation). The second configuration, called “unbounded” diffusion (right figure in Scheme 1), is the one in which the wall at *x* = *L* is an electroactive element that collects the species arriving from the electrode surface and converts them totally or partially to their initial values in the inverse faradaic process that takes place at *x* = 0. In agreement with Scheme 1, a complete conversion will be assumed (see below). Note that this last situation differs from the response of an homogeneous catalytic mechanism (EC′) in the fact that the regeneration of species R takes place only at *x* = *L* and not in the solution (see refs 41 and 42 ).

**Scheme 1:**
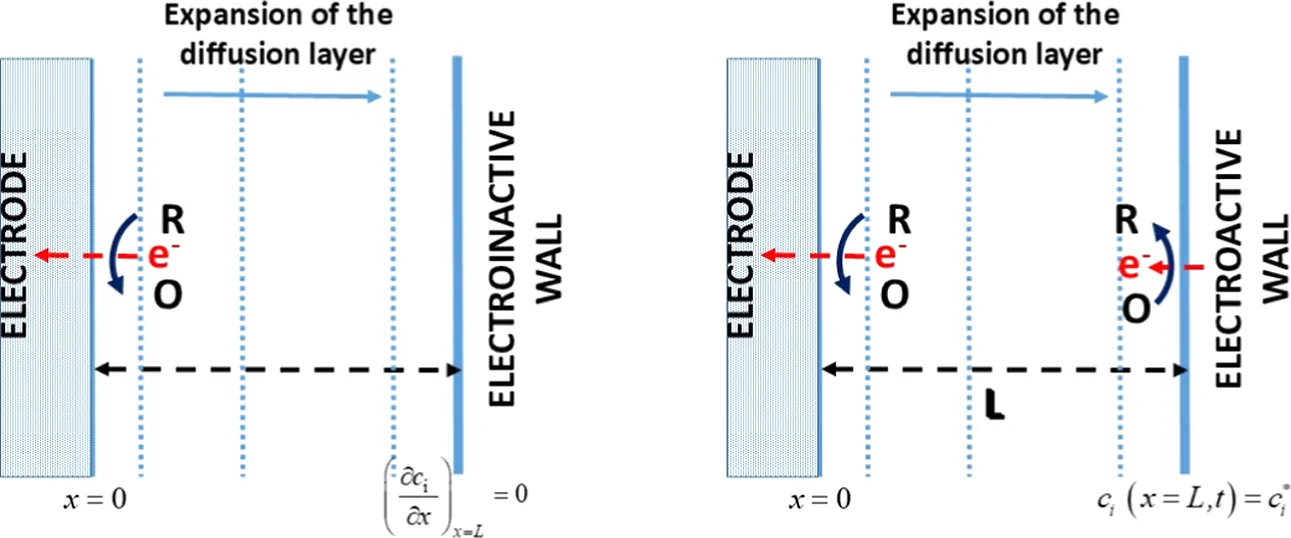
Bounded (Left) and Unbounded (Right) Diffusive Fields Taking Place at a Region Located at 0 ≤ *x* ≤ *L*; The Electrode Surface is Located at *x* = 0

Here, a unified treatment for both situations is presented to rationalize the Chronoamperometry and Normal Pulse Voltammetry (NPV) responses of redox probes under FD conditions, including as limiting cases the SID and TL situations.

By assuming that the diffusion coefficients of both species are equal, i.e., *D*_R_ = *D*_O_ = *D*, and that the mass transport is only by linear diffusion, the following differential equation describes the time and spatial variation of both species (second Fick’s law)

$$\frac{\partial c_{i}^{B/U}}{\partial t}=D\left(\frac{\partial^{2}c_{i}^{B/U}}{\partial x^{2}}\right)\;,i=R,O$$ 1

Superscripts “B” and “U” in these expressions refer to the bounded or unbounded mass transport conditions, respectively.

This differential equation system has the following boundary value problem

For *t* = 0, 0 ≤ *x* ≤ *L*,

$$c_{i}^{B/U}\left(x,t\right)=c_{i}^{*},i=R,O$$ 2

For *t* > 0, *x* = 0

3 $${\left(\frac{\partial c_{R}^{B/U}}{\partial x}\right)}_{x=0}=-{\left(\frac{\partial c_{O}^{B/U}}{\partial x}\right)}_{x=0}$$

4 $$\frac{I^{B/U}}{FA}=D{\left(\frac{\partial c_{R}^{B/U}}{\partial x}\right)}_{x=0}=k_{ox}c_{R}^{s,B/U}-k_{red}c_{O}^{s,B/U}$$

with *I* ^B/U^ being the current, and $c_{i}^{s,B/U}$ (i = R, O) the surface concentrations (at *x* = 0) of species i.

For bounded conditions it is fulfilled that, for *x* = *L*

5 $${\left(\frac{\partial c_{R}^{B}}{\partial x}\right)}_{x=L}={\left(\frac{\partial c_{O}^{B}}{\partial x}\right)}_{x=L}=0$$

whereas for unbounded conditions it is fulfilled that for *x* = *L*

$$c_{i}^{U}\left(x=L,t\right)=c_{i}^{*},i=R,O$$ 6

The boundary condition ([Disp-formula eq5]) establishes null flux of the different electroactive species at the wall located at *x* = *L*,^29,39,43,46^ whereas condition ([Disp-formula eq6]) indicates the complete faradaic conversion of the depleted species toward their bulk values at *x* = *L*.^29,30^

The expression of the current obtained for the application of a constant potential has been deduced in Section S2 of SI. If we assume that the total time of application of the potential waveform is divided into *m* intervals of length Δτ, such that *t* = *m*Δτ, the current can be written in the way

$$I_{m}^{B/U}=\frac{I_{0}-k_{T}\overset{m-1}{\underset{j=1}{\sum }}\left(I_{j}{ikernel}^{B/U}\left(m-j\right)\right)}{1+k_{T}{ikernel}^{B/U}\left(0\right)},m=1,2,...$$ 7

where

8 $$I_{0}=I\left(t\to 0\right)=FAc_{R}^{*}\left(k_{ox}-\mu k_{red}\right)$$

9 $$k_{T}=k_{ox}+k_{red}$$

10 $$\mu =\frac{c_{O}^{*}}{c_{R}^{*}}$$

$${ikernel}^{B}\left(z\right)=\{\begin{array}{l} 2\sqrt{\frac{\Delta \tau }{\pi D}}\left(\sqrt{z+1}-\sqrt{z}\right),\text{if}\;\left(Dz\Delta \tau /L^{2}\right)\leq 0.12\\ \Delta \tau +\frac{2L^{2}}{\pi^{2}D}\left(e^{-(\frac{\pi^{2}D}{L^{2}})z\Delta \tau }-e^{-(\frac{\pi^{2}D}{L^{2}})\left(z+1\right)\Delta \tau }+\frac{1}{4}\left(e^{-(\frac{4\pi^{2}D}{L^{2}})z\Delta \tau }-e^{-(\frac{4\pi^{2}D}{L^{2}})\left(z+1\right)\Delta \tau }\right)\right)\\ \text{if}\;\left(Dz\Delta \tau /L^{2}\right)>0.12 \end{array}$$ 11

$${ikernel}^{U}\left(z\right)=\{\begin{array}{l} 2\sqrt{\frac{\Delta \tau }{\pi D}}\left(\sqrt{z+1}-\sqrt{z}\right),\text{if}\;\left(Dz\Delta \tau /L^{2}\right)\leq 0.12\\ \frac{8L}{\pi^{2}D}\left(e^{-(\frac{\pi^{2}D}{4L^{2}})z\Delta \tau }-e^{-(\frac{\pi^{2}D}{L^{2}})\left(z+1\right)\Delta \tau }+\frac{1}{9}\left(e^{-(\frac{9\pi^{2}D}{4L^{2}})z\Delta \tau }-e^{-(\frac{9\pi^{2}D}{L^{2}})\left(z+1\right)\Delta \tau }\right)\right)\\ \text{if}\;\left(Dz\Delta \tau /L^{2}\right)>0.12 \end{array}$$ 12

The SID limit can be obtained from the expressions of ikernel^B/U^ in eqs 11 and 12 by imposing *L* → ∞

13 $${ikernel}^{SID}\left(z\right)={{ikernel}^{B/U}|}_{L\to \infty }\left(z\right)=2\sqrt{\frac{\Delta \tau }{\pi D}}\left(\sqrt{z+1}-\sqrt{z}\right)$$

In agreement with eq 7, the current is obtained in an iterative way. *k*_T_ takes a constant value for the application of a constant potential, and it fixes the potential dependence of the response (see eq 9 and Figure S1 in Section S1), whereas functions ikernel^B/U^ establish the time dependence of the current for the operating mass transport mode (B, U or SID).

The expression for the current given in eq 7 will be applied to Chronoamperometry (CA) and Normal Pulse Voltammetry (NPV). The response will be considered as a single experiment (CA) or a series of independent experiments (in the sense that for all the potentials applied the “initial” conditions given by eq 2 hold, NPV).

From the general solution for the current given in eq 7 it is possible to obtain the following limits:

(a) Semi-infinite diffusion (SID) limit (*L* → ∞)

The current given in eq 7 can be used with ikernel^SID^ (see eq 13). Moreover, in this case it is possible to obtain an explicit expression for the current.^1,3^ This well-known expression is the following

14 $$I^{SID}\left(t\right)=FAc_{R}^{*}\left(k_{ox}-\mu k_{red}\right)\frac{1}{k_{T}}\sqrt{\frac{D}{\pi t}}F\left(\chi \right)$$

where

15 $$F\left(\chi \right)=\sqrt{\pi }\chi e^{\chi^{2}}erfc\left(\chi \right)$$

16 $$\chi =k_{T}\sqrt{\frac{t}{D}}$$

and *erfc*(*x*) in eq 15 is the complementary error function (*erfc*(*x*) = 1 – *erf*(*x*)).^3,55^ eq 14 leads to the following limit for reversible processes (i. e., fast kinetics, conditional rate constant *k* ^0′^ ≫ 1 cm s^–1^)^1,3,56^

17 $$I_{reversible}^{SID}\left(t\right)=FAc_{R}^{*}\frac{e^{\eta }-\mu }{1+e^{\eta }}\sqrt{\frac{D}{\pi t}}$$

with η = *F*(*E* – *E* ^0′^)/*RT*.

(b) Thin-layer (TL) limit (*L* → 0)

In a contrary way, when the thickness of the solution layer tends to zero in eq 11, from eq 7 the following expression, corresponding to the TL limit for the bounded case is deduced

$$I^{B,TL}\left(t\right)=Q_{F}\left(\frac{k_{ox}}{L}-\mu \frac{k_{red}}{L}\right)e^{-\left(\frac{k_{T}}{L}\right)t}$$ 18

where

19 $$Q_{F}=FAc_{R}^{*}L$$

Equation 18 has an expression formally identical to that obtained for a diffusionless system (i. e., for a confined electroactive species forming a redox monolayer^3,57,58^ ), by changing (*k*_i,solution_/*L*) = *k*_i,surface_, i = R, O, and *Q*_F_ by *Q*_F,surface_ = *FA*Γ_T_, with Γ_T_ being the total excess, by the expression given in eq 19.

In the case of the unbounded configuration, the TL limit deduced from eqs 7 and 12 is

20 $$I^{U,TL}=\frac{FADc_{R}^{*}}{L}\frac{\left(k_{ox}\left(\frac{L}{D}\right)-\mu k_{red}\left(\frac{L}{D}\right)\right)}{1+k_{T}\left(\frac{L}{D}\right)}$$

which corresponds to a stationary response. For a fast kinetics case, eq 20 simplifies to

21 $$I_{reversible}^{U,TL}=\frac{FADc_{R}^{*}}{L}\frac{e^{\eta }-\mu }{1+e^{\eta }}$$

with (*FAD* $c_{R}^{*}$ )/*L* being the maximum stationary current that can be obtained under unbounded conditions, independently of the reversibility degree of the charge transfer reaction.^29^

Note that the above expressions for the current do not depend on the kinetic formalism used. Therefore, they are valid independently of the election of Butler–Volmer or Marcus–Hush–Chidsey expressions for the rate constants.

General expression for the current, given by eq 7, becomes into any of the particular limits given above depending on the experimental conditions (mass transport operating mode, time, thickness of the diffusion layer, diffusion coefficient and rate constant).

## Results and Discussion

### Chronoamperometry

The simplest electrochemical response of a given redox probe is that corresponding to the current–time curve obtained for the application of a single constant potential (eq 7). In the analysis of the chronoamperometric response, it will be considered the influence of the rate constant value (in both Butler–Volmer, BV, and Marcus–Hush–Chidsey, MHC, formalisms) for different values of the length of the diffusion field *L* between the SID and TL limits. Both bounded and unbounded configurations will be discussed.

Figure 1 shows the current–time responses obtained under bounded (A) and unbounded (B) conditions, corresponding to a charge transfer process with a conditional rate constant *k* ^0′^ = 10^–3^ cm s^–1^ for a symmetrical BV (α = 0.5, solid lines) or MHC formalism (with an asymmetry parameter γ = 0 and λ = 1 eV, dashed lines), calculated from eqs 7–13 for different values of *L* in microns. The applied potential versus the formal potential has been Δ*E* = *E*_applied_ – *E* ^0′^ = 0.200 V, so kinetic effects can be observed in the current–time curves. The response corresponding to SID conditions (eq 14) has also been included for comparison (gray lines).

**Figure 1:**
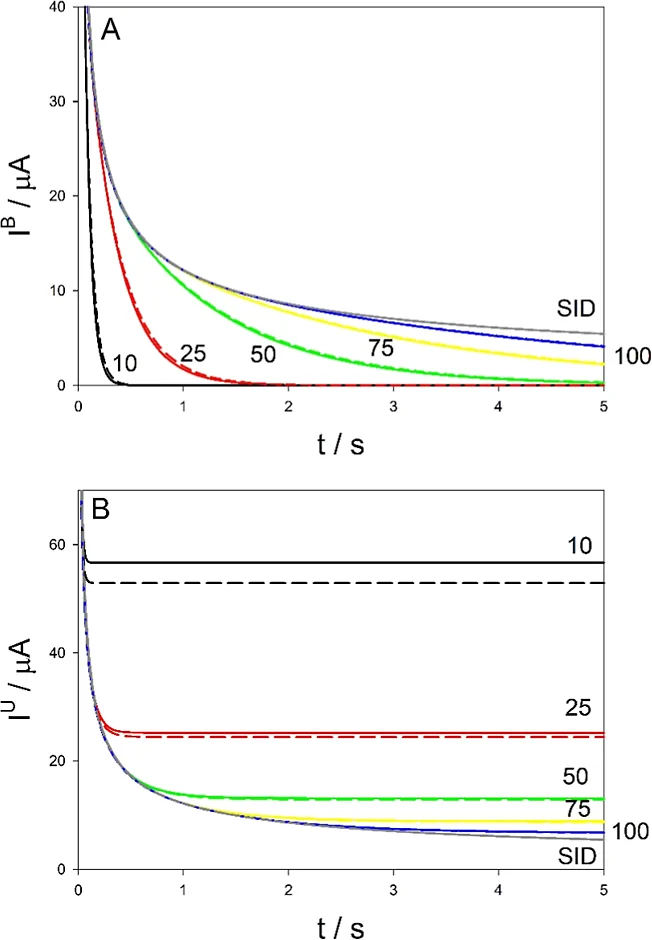
Influence of *L* on the chronoamperometric *I* ^B/U/SID^–*t* curves for bounded (A) and unbounded (B) mass transport modes calculated by using eqs 7–13 for symmetrical BV (α = 0.5, solid lines) and MHC (λ = 1 eV, γ = 0, dashed lines) formalisms. *k* ^0′^ = 10^–3^ cm s^–1^, *D* = 10^–5^ cm^2^ s^–1^, Δ*E* = *E*_applied_ – *E* ^0′^ = 0.200 V, $c_{R}^{*}$ = 1.0 mM, *A* = 0.0707 cm^2^. The values of *L* (in microns) are given in the Figure. The curves corresponding to SID conditions (gray lines) have been calculated from eq 14 and included for comparison.

Under bounded conditions (Figure 1A), the current decreases more rapidly the thinner the solution layer, indicating a faster depletion of the electroactive species. This behavior is analogous to that observed for fast kinetics.^29^ For the rate constant chosen here, no significant differences are apparently observed between BV and MHC formalisms. It is interesting to observe that, as *L* decreases (below 100 μm for the *D* value chosen), the current shows an exponential dependence on time of the form $I^{B}\left(t\right)=constant\times e^{-f\left(L\right)k_{T}t}$ (see Figure S2), with *f*(*L*) being a function which changes depending on the value of the distance *L*. Thus, for the limiting TL behavior, *f*(*L*) = 1/*L* (see eq 18). This exponential dependence gives rise to linear logarithmic plots for the currents with higher slopes (in absolute value) as *L* decreases, where the differences between BV and MHC are more evident for *L* ≤ 25 μm for the *D* value chosen here (see Figure S2).

For unbounded conditions (Figure 1B), the current increases and becomes stationary as *L* decreases (this stationary value is attached at lower times the thinner the solution layer is), in an analogous way to that observed for fast kinetics.^29^

The stationary currents in Figure 1B have values that increase with (1/*L*) in agreement with eq 20, with this value depending on the rate constants. Moreover, as *L* decreases, the differences between BV and MHC formalisms are more evident (see the curve with 10 μm).

It is clear from Figure 1 that, when comparing the degree of influence of BV and MHC formalisms on the *I* ^B/U^–*t* curves as the thickness of the diffusion layer decreases, bounded and unbounded configuration show a different behavior. Thus, for the bounded case, no significant differences are observed between BV and MHC models, whereas in the unbounded case, the discrepancies between both kinetic formalisms are more evident for the smaller *L* values. This different influence of the mass transport mode on the overall kinetic effects on the current is based on the dependence of the current behavior on the dimensionless rate constant, that incorporate both the operating mass transport for a given experimental configuration and redox kinetics effects in a single expression.^1,3^ For a SID configuration, the expression of the dimensionless rate constant is

22 $${\mathrm{k̅}}_{SID}^{0^{\prime }}=k^{0^{\prime }}\sqrt{\frac{\tau }{D}}$$

with τ being the experimental time.^3^

In agreement with the theoretical analysis carried out in Section S2 of SI, the TL limit of the dimensionless rate constants is different for the bounded and unbounded configurations (see eqs S38 and S41 of Section S2)­

23 $$\begin{array}{l} {\mathrm{k̅}}_{B,TL}^{0^{\prime }}=\frac{k^{0^{\prime }}\tau }{L}\\ {\mathrm{k̅}}_{U,TL}^{0^{\prime }}=\frac{k^{0^{\prime }}L}{D} \end{array}$$

From the SID and TL limits given in eqs 22–23, it is convenient to define general expressions for the overall dimensionless rate constants for both mass transport modes

24 $$\begin{array}{l} {\mathrm{k̅}}_{B,FD}^{0^{\prime }}=k^{0^{\prime }}\left(\sqrt{\frac{\tau }{D}}+\frac{\tau }{L}\right)\\ {\mathrm{k̅}}_{U,FD}^{0^{\prime }}=\frac{k^{0^{\prime }}}{\sqrt{\frac{D}{\tau }}+\frac{D}{L}} \end{array}\}$$

In line with eq 24, the value of ${\mathrm{k̅}}_{B,FD}^{0^{\prime }}$ always increases when moving from SID to TL conditions whereas the opposite behavior is observed for ${\mathrm{k̅}}_{U,FD}^{0^{\prime }}$ . This behavior can be seen in Figure 2, and it implies that a given charge transfer reaction behaves as more reversible for a bounded configuration whereas the opposite behavior should be observed for unbounded conditions, this effect on reversibility being greater the lower the value of *L*. The attachment of pure TL conditions (eq 23) depends on the experimental conditions. Thus, for values of *L* ≤ 0.3 μm for a diffusion coefficient *D* = 10^–5^ cm^2^ s^–1^ and a total time of 1.0 s the dimensionless rate constants take their TL limiting values with an error below 1% (in general, this limit is reached for values of $\sqrt{D\tau }\geq 100L$ ).

**Figure 2:**
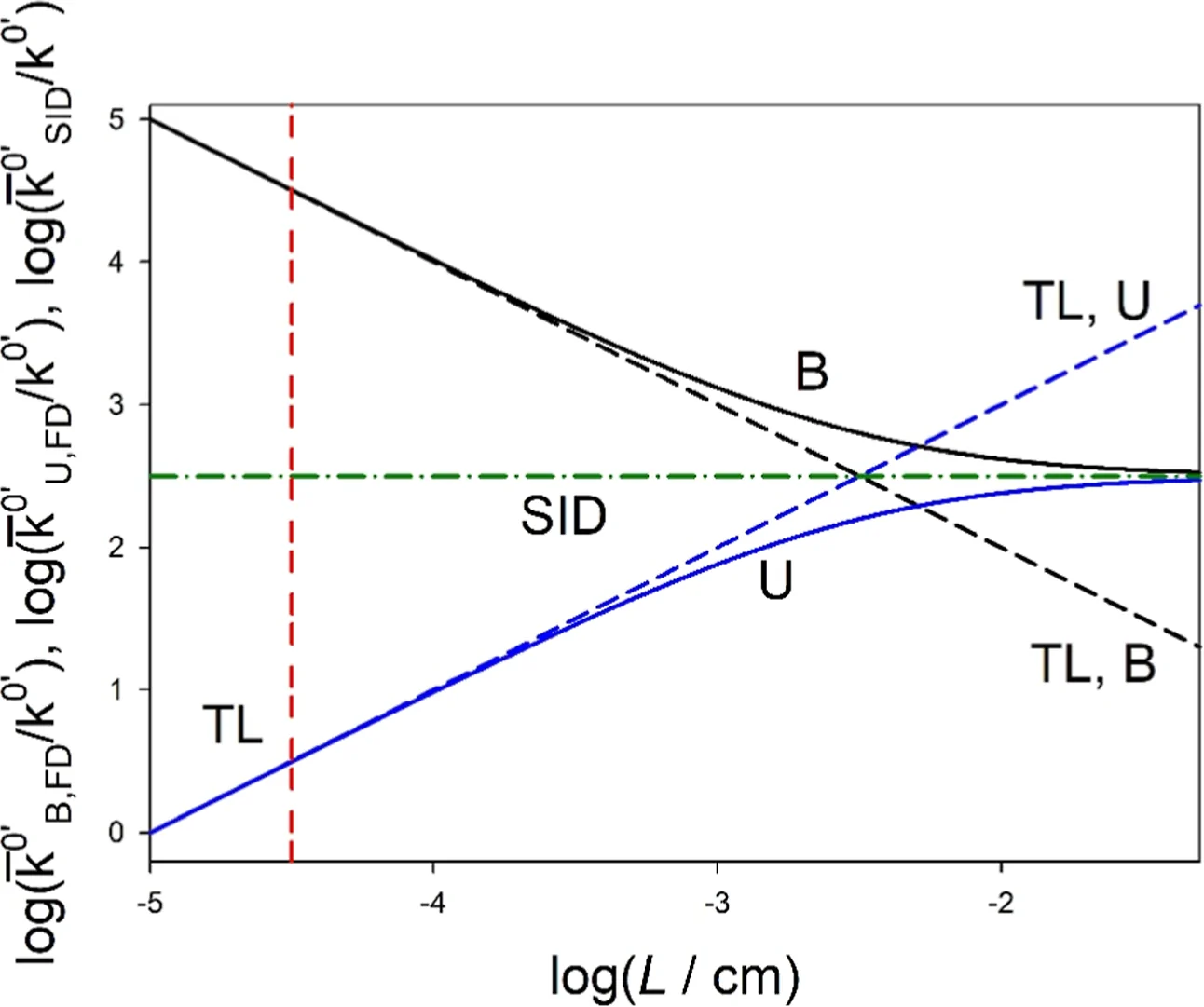
Evolution of the logarithm of the dimensionless rate constants versus log­(*L*) for bounded ( ${\mathrm{k̅}}_{B,FD}^{0^{\prime }}/k^{0^{\prime }}$ , black lines) and unbounded ( ${\mathrm{k̅}}_{U,FD}^{0^{\prime }}/k^{0^{\prime }}$ , blue lines) diffusion calculated from eqs 22–24 for a time τ = 1.0 s and a diffusion coefficient *D* = 10^–5^ cm^2^ s^–1^. Vertical dashed red line marks the pure TL region.

The variation in the length of the diffusive field *L* affects both to the dimensionless rate constant (in agreement with eq 24) and to the dimensionless distance parameter $\lambda =L/\sqrt{D\tau }$ (see Section S2, eqs S38 and S41). In order to analyze the effect of redox kinetics in the current–potential response for a fixed value of *L*, Figure 3 shows the current–time curves obtained for bounded (A) and unbounded (B) conditions corresponding to the application of an applied potential versus the formal potential of Δ*E* = *E*_applied_ – *E* ^0′^ = 0.250 V, calculated for different values of the rate constant *k* ^0′^ and a fixed distance of *L* = 25 μm in both BV (solid lines) and MHC (dashed lines) formalisms. The curves obtained under SID conditions have been included in inner Figure 3a for comparison.

**Figure 3:**
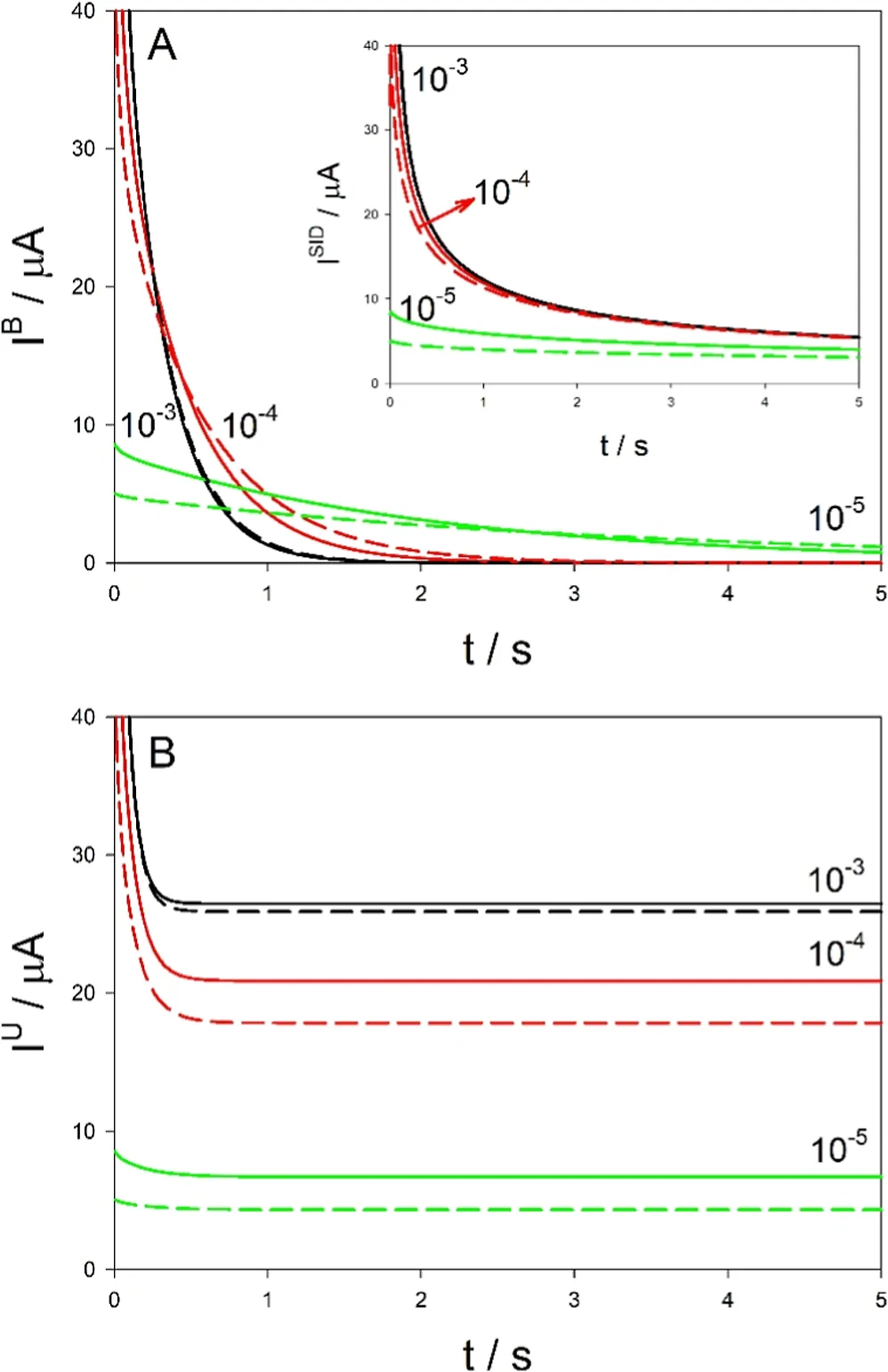
Influence of the rate constant *k* ^0′^ on the *I* ^B/U/SID^–*t* curves for bounded (A), and unbounded (B) mass transport modes, calculated by using eqs 7–13 for BV (α = 0.5, solid lines) and MHC (λ = 1 eV, γ = 0, dashed lines) formalisms. *L* = 25 μm, *D* = 10^–5^ cm^2^ s^–1^, Δ*E* = *E*_applied_-*E* ^0′^ = 0.250 V, $c_{R}^{*}$ = 1.0 mM, A = 0.0707 cm^2^. The values of *k* ^0′^ (in cm s^–1^) are given in the Figure.

In all the cases, the decrease of the rate constant leads to a decrease in the current for this applied potential due to a higher resistance of the charge transfer (that is, to a higher difficulty in kinetic terms for the charge transfer process). Differences between BV and MHC are observed for *k* ^0′^ ≤ 10^–3^ cm s^–1^, leading to smaller currents in the MHC case since the effective rate constants are smaller (see Section S1). Moreover, in the bounded case (Figure 3A), there appear crosses between the chronoamperograms obtained for the different rate constants. These crosses arise from the fact that the decrease of the rate constant results in a slower depletion of the electroactive species and consequently prolongs the current response, whereas the finite diffusive field leads to a faster depletion of the electroactive species. At shorter times, the dominant effect is the kinetic one (higher current for higher rate constants), but this changes at longer times, with the mass transport effect dominating the time evolution of the current. This can also be seen in the logarithm currents of Figure S3, where the differences between both kinetic formalisms are also evident.

For the unbounded case shown in Figure 3B, the current becomes stationary for the different rate constants, and this value depends on the kinetics in line with eq 20. Differences between BV and MHC are more evident than in the bounded case.

The stationary currents obtained for the unbounded configuration increase their value with the rate constant. The maximum value for these currents is that corresponding to fast kinetics (see eq 21), and to reach this limit, higher values of the rate constant are required as *L* decreases in line with the previous discussion. This behavior can also be seen in Figure S4, which shows the evolution of *I* ^U^ with *k* ^0′^ for different values of *L*.

### Normal Pulse Voltammetry

The electrochemical technique Normal Pulse Voltammetry is a single pulse technique in which a series of constant potentials of the same duration is applied to the electrode in such a way that between the application of two of them, the initial conditions are restored. Therefore, it can be considered that the response of each potential in the sequence is independent of the previous ones. Moreover, the chronoamperometric current obtained for each potential is sampled at fixed time and plotted versus the applied potential.^3^ For achieving “initial” conditions previous to the application of a given potential of the sequence, a rest potential where the electroactive species is returned to its bulk value is applied during enough time. The current–potential plots obtained under SID conditions are typically sigmoidal, with the plateau current being coincident with the mass transport limiting current for the operating mass transport regime.^3^

The NPV responses of confined species for both transport modes will be analyzed to get an adequate comprehension of the influences that determine the overall behavior of the current. This technique can be applied to the characterization of the redox kinetics behavior under finite conditions, and it provides responses very sensitive to the kinetics and mass transport mode. Moreover, under certain circumstances, it could be possible to determine accurately the rate constants and characteristics parameters (formal potential, diffusion coefficients, ...) for a practical situation in which the response is affected by a complex combination of time dependences.

Figure 4 shows the effect of the decrease of the thickness of the solution layer *L* on the NPV curves obtained for a given rate constant (*k* ^0′^ = 10^–3^ cm s^–1^) for bounded (A) and unbounded (B) conditions when symmetrical BV (α = 0.5, solid lines) and MHC (γ = 0 and λ = 1 eV, dashed lines) are considered. The values of *L* are in the range 10–50 μm and the SID response has been included for comparison (see gray line).

**Figure 4:**
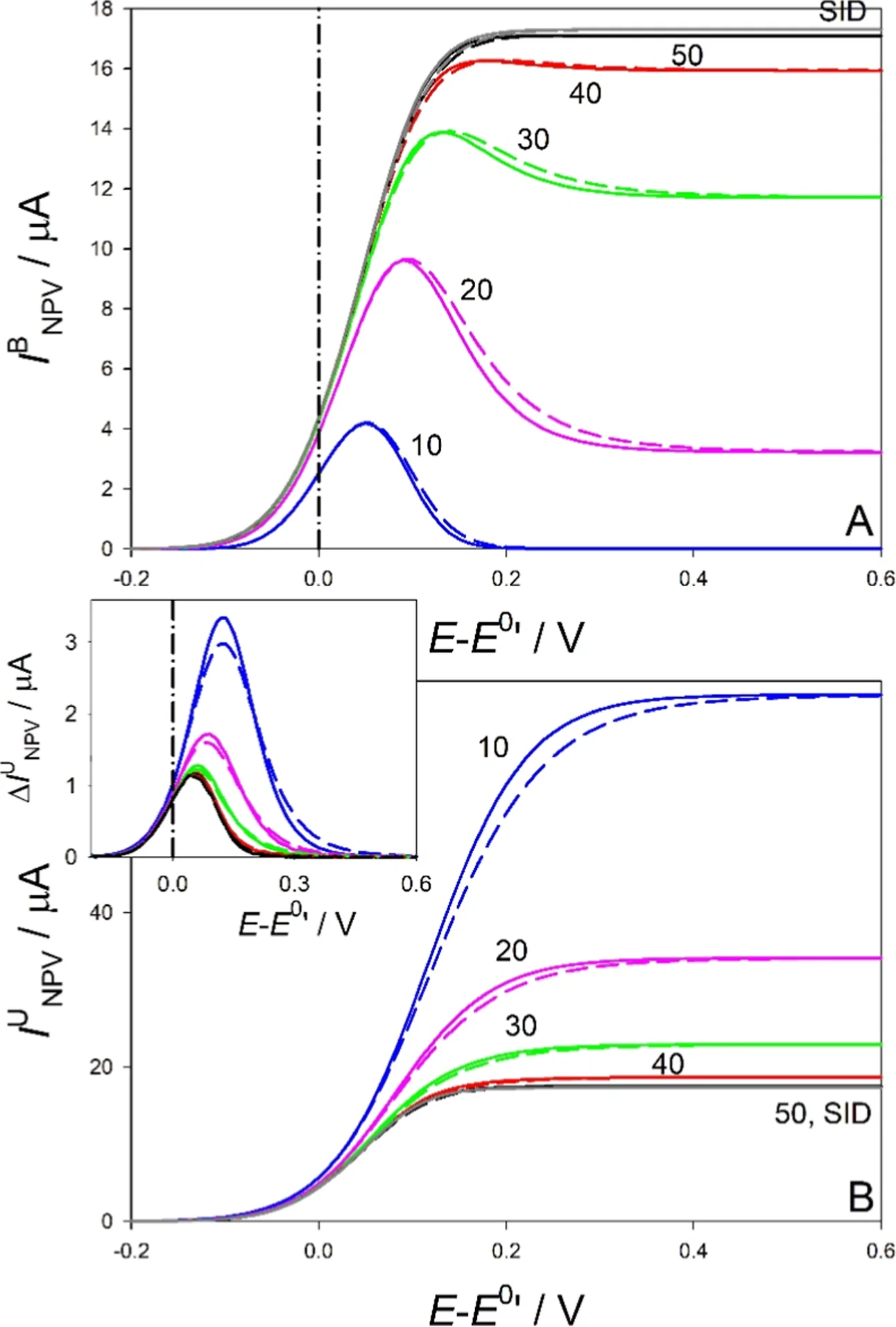
Influence of *L* in the NPV curves for bounded (A) and unbounded (B) mass transport modes calculated by using eqs 7–12 for BV (α = 0.5, solid lines) and MHC (λ = 1 eV, γ = 0, dashed lines) formalisms. *k* ^0′^ = 10^–3^ cm s^–1^, *D* = 10^–5^ cm^2^ s^–1^, Δ*E* = 10 mV, τ = 0.5 s, $c_{R}^{*}$ = 1.0 mM, A = 0.0707 cm^2^. The values of *L* (in microns) are given in the Figure. The curves corresponding to SID conditions (eq 14, gray lines) have been included for comparison. Inner (B): ${\Delta I}_{U}^{NPV}$ = $I_{U}^{NPV}$ ­(*E*_*j*+1_) – $I_{U}^{NPV}$ ­(*E*_*j*_) vs *E* obtained from curves in Figure 4B. Vertical black dashed-dotted line marks the location of the formal potential.

Under bounded conditions (Figure 4A), when we move from SID to TL conditions, the NPV current changes its shape and it shows an increasingly pronounced peak as *L* decreases. The appearance of this peak is a consequence of the change in the controlling process of the response (from redox kinetics to availability of redox species, see the crosses in the chronoamperometric responses of Figure 3A). After the peak, the current reaches a plateau for positive enough potentials corresponding to the limiting current for the corresponding value of *L*, which is independent of the value of the kinetic rate constant and it is given by^29^

25 $$I_{\lim}^{B}=FAc_{R}^{*}\sqrt{\frac{D}{\pi \tau }}\left(1+2\sum_{j=1}^{\infty }{\left(-1\right)}^{j}e^{-j^{2}L^{2}/\left(D\tau \right)}\right)$$

The peak potential shifts toward the formal potential of the redox process (see dashed dotted line) as *L* decreases, and the limiting current tends to zero for potentials above 0.2 V for *L* ≤ 10 μm for the time pulse considered in these curves (τ = 0.5 s). Concerning the differences between BV and MHC formalisms, for the *k* ^0′^ value chosen here, it is clear that they are small and located around the peak current with MHC curves showing a broader peak, especially for 20 μm (see pink dashed curve in Figure 4A).

For unbounded conditions (Figure 4B), all the curves are sigmoidal, with the limiting current obtained at positive enough potential being independent of the kinetic rate constant and being given by^29^

26 $$I_{\lim}^{U}=FAc_{R}^{*}\sqrt{\frac{D}{\pi \tau }}\left(1+2\sum_{j=1}^{\infty }e^{-j^{2}L^{2}/\left(D\tau \right)}\right)$$

which for small enough values of *L*, simplified to

27 $$I_{\lim}^{U,TL}=\frac{FADc_{R}^{*}}{L}$$

This transport mode gives rise to an increase of the apparent redox irreversibility of the responses as *L* decreases, in line with Figure 2. The half-wave potential of the NPV responses moves away from the formal potential in an opposite trend to that observed for bounded conditions, as shown in the inner Figure 4B where the difference between two consecutive NPV current values, Δ*I* ^U^, has been plotted vs the applied potential. The peak potential observed in these differential curves coincides with the half wave potential in Figure 4B. Moreover, MHC curves spread through wider potential zones than the BV ones such that the Δ*I* ^U^–*E* curves show broader and smaller peaks.

Figure 5 shows the effect on the NPV curves of the decrease of the kinetic rate constant for a fixed value of *L* = 25 μm for both transport modes and both kinetic formalisms. The response obtained for fast kinetics (FK curve) has been included for comparison (gray lines in Figure 5).

**Figure 5:**
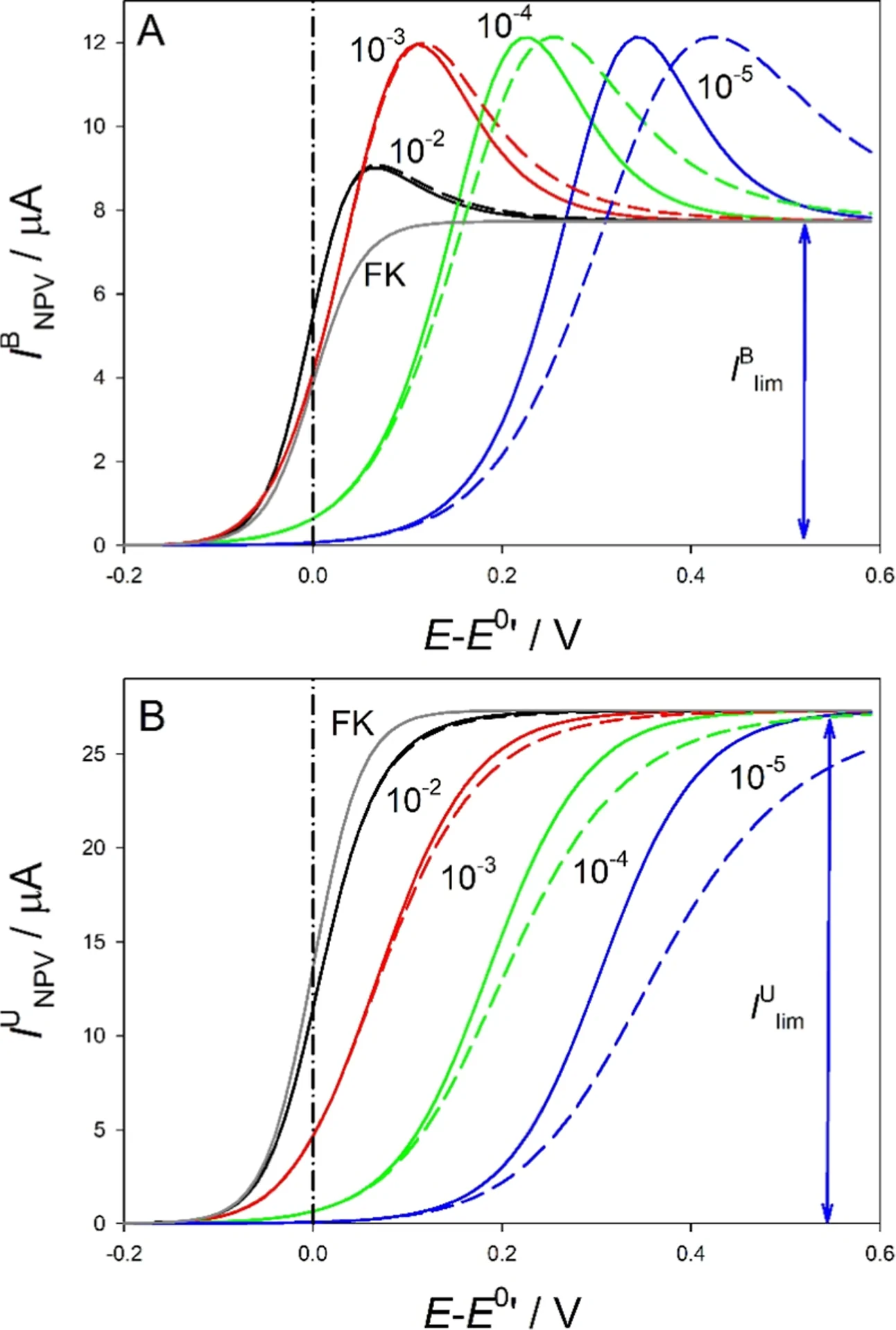
Influence of *k* ^0′^ in the NPV curves for bounded (A) and unbounded (B) mass transport modes calculated by using eqs 7–13 for BV (α = 0.5, solid lines) and MHC (γ = 0, λ = 1 eV, dashed lines) formalisms. *L* = 25 μm, *D* = 10^–5^ cm^2^ s^–1^, Δ*E* = 10 mV, τ = 0.5 s, $c_{R}^{*}$ = 1.0 mM, A = 0.0707 cm^2^. The values of *k* ^0′^ (in cm s^–1^) are given in the Figure. The response corresponding to a reversible process (fast kinetics, FK, gray lines) is shown for comparison. Vertical black dashed-dotted line marks the location of the formal potential.

In the case of the bounded NPV curves shown in Figure 5A, the shape of the response is sigmoidal for a fast kinetic process, and it develops a growing peak as *k* ^0′^ decreases. The characteristic features of this peak (peak current, $I_{NPV,peak}^{B}$ , peak potential, $E_{NPV,peak}^{B}$ , and half-peak width, *W*_1/2_) can be seen in Figure 6A. The NPV peak reaches its maximum height for *k* ^0′^ = 10^–3^ cm s^–1^, a limiting value for the fully irreversible behavior. As the rate constant decreases and the process behaves as more irreversible, the NPV curve is shifted toward more positive potentials, but the height of the peak remains unaltered. Both trends can be seen in Figure 6B, which shows the evolution of peak parameters as a function of the logarithm of the rate constant log­(*k* ^0′^) (see eq 24). Therefore, the peak height for fully irreversible processes only depends on the value of *L* and not on the rate constant neither on the kinetic formalism (see inner Figure 6B). The peak potential moves toward the formal potential values for processes close to a fast kinetics behavior but, when irreversibility becomes relevant, it moves away from *E* ^0′^, much more markedly in the case of MHC formalism. Finally, the half peak width increases as the rate constant decreases, attaining a constant value for the BV case whereas it always increases as the process becomes more irreversible for the MHC formalism (see Figures 5A and 6B).

**Figure 6:**
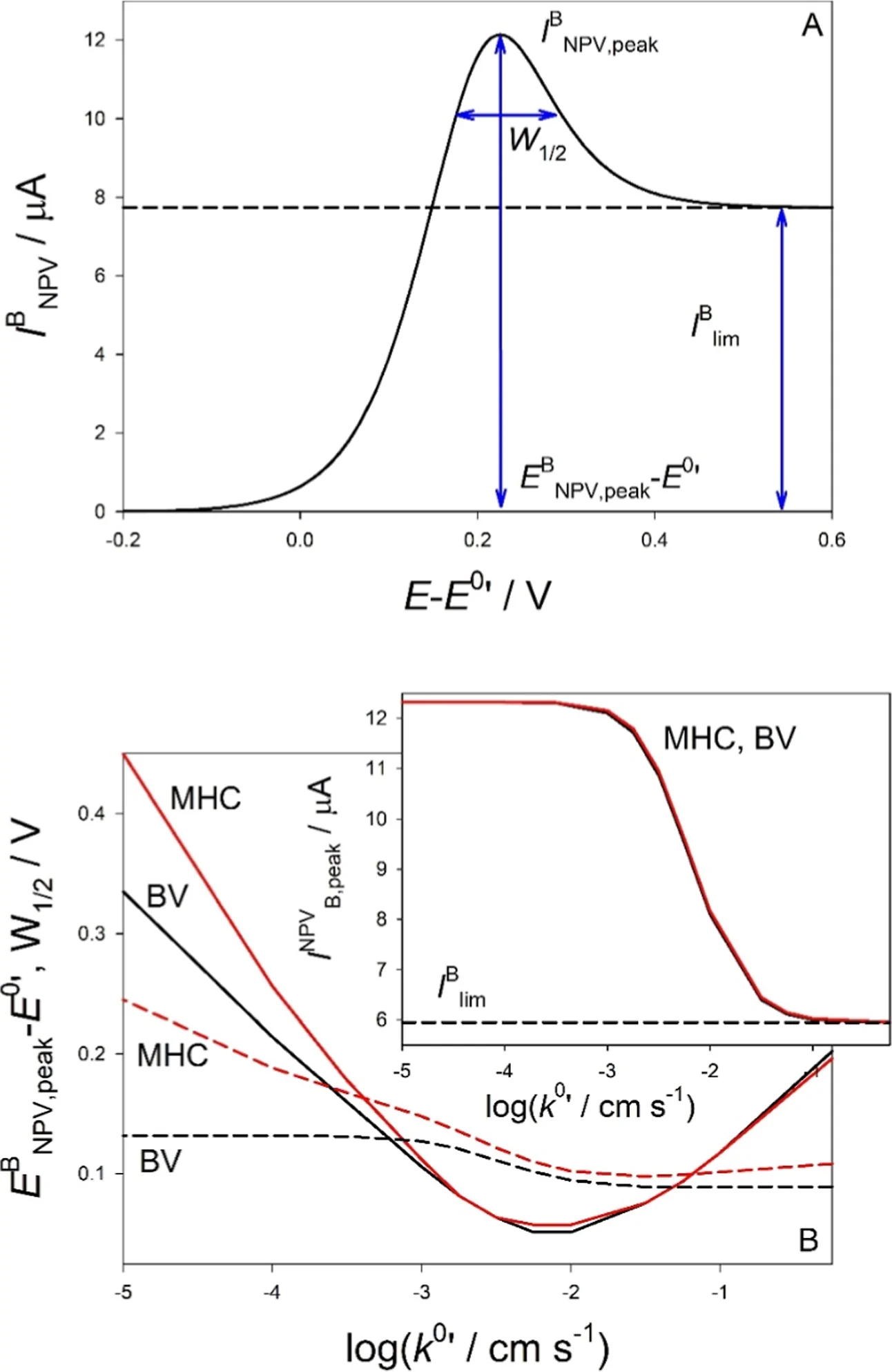
(A) Characteristic features of a NPV curve for a quasi-reversible charge transfer process under bounded conditions. (B) Evolution of the peak potential (solid lines), half-peak width (dashed lines) and peak current (inner Figure) with log­(*k* ^0′^/cm s^–1^). τ = 0.2 s, *A* = 0.0707 cm^2^, $c_{R}^{*}$ = 1.0 mM, *L* = 10 μm, *D* = 10^–5^ cm^2^ s^–1^, *T* = 298 K. Black and red lines correspond to BV (α = 0.5) and MHC (γ = 0, λ = 0.75 eV), formalisms, respectively.

The NPV responses obtained for the unbounded configuration are shown in Figure 5B. These curves are sigmoidal in all the cases, and they move toward more positive potentials as *k* ^0′^ decreases, with the differences between BV and MHC responses becoming more evident for fully irreversible processes. The half wave potential of these curves can be obtained from eq 20

28 $$k_{ox}^{1/2}-k_{red}^{1/2}=\frac{D}{L}$$

with $k_{ox/red}^{1/2}$ being the corresponding rate constant at the half wave potential. For the symmetrical BV case (α = 0.5), eq 28 can be solved, with *E* ^1/2^ being

$$E^{1/2}=E^{0^{\prime }}+\frac{2RT}{F}\ln\left(\frac{1+\sqrt{1+4{\left({\overset{¯}{k}}_{U,T\,\;\,L}^{0^{\prime }}\right)}^{2}}}{2{\overset{¯}{k}}_{U,T\,\;\;L}^{0^{\prime }}}\right)$$ 29

with ${\mathrm{k̅}}_{U,TL}^{0^{\prime }}$ given by eq 23.

Finally, the influence of the symmetry of the charge transfer (through the value of charge transfer coefficient α for a BV approach, or through the asymmetry parameter γ for a MHC one), has been considered in Figure S5 of Section S3 for a *k* ^0′^ = 10^–3^ cm s^–1^, and values of α in the range (0.3, 0.7) and γ in the range (−0.5, 0.85). As can be seen in this figure, for the bounded case, the increase of charge transfer coefficient α (or its equivalent increase in γ), leads to a broadening of the NPV peak and a shift of the peak position toward more positive potentials. The effect of the symmetry of the charge transfer process in the NPV peak height is less relevant (see Figure S5A). In the unbounded case, the increase of charge transfer coefficient α (or its equivalent increase in γ), leads to a decrease of the slope of the rising region of these curves, together with a shift of the half wave potential toward more positive potentials (see Figure S5B).

### Experimental Results

The methodology described in previous sections has been applied to the analysis of the electrochemical response of a given redox probe. We have focused on Normal Pulse Voltammetry since the chronoamperometric responses at short times (for which the currents are high) could present some ohmic distortion (see Sections S4–S5 of SI and refs 29, 59 and 60 ).

As proof of concept to analyze the potential applicability of NPV technique in the identification and quantitative study of quasi-reversible processes, it has been analyzed the redox behavior of radical 4-Carboxy-2,2,6,6-tetramethylpiperidine 1-oxyl (radical 4-Carboxy-TEMPO, 4CT). TEMPO radical has special relevance in different fields as are electrosynthesis (especially for the oxidation of alcohol groups) or electric energy storage (in organic or aqueous–organic redox flow batteries).^61–63^ Although the oxidation of the nitrosyl group is assumed to be fast,^61^ there can be kinetic effects when the TEMPO backbone has functional groups that can affect to the electronic density. In this case, the carboxy group present in 4CT, due to its electron-withdrawing nature, could destabilize the product of the oxidation reaction, slowing it down. There is scarce information relative to the rate constant for the oxidation of TEMPO derivatives, and all the existing studies have been carried out under SID conditions. For the case of 4CT, in ref,^64^ the rate constant is obtained by assuming a BV formalism and using a Rotating Disc Electrode in a 0.5 M KCl aqueous media, being *k* ^0′^ = 2.25 × 10^–3^ cm s^–1^ (for a charge transfer coefficient α = 0.5).

Two different FD cells with an adaptable diffusive field length were used. In both cases the working electrode (a Boron-Doped Diamond, BDD, disc electrode of 1.5 mm radius) was fixed to a piezoelectric device that could move up and down with micrometre-level resolution, enabling *L* value to be adjusted.

The FD cell for the bounded configuration is a 20 × 20 × 8 mm square piece of PVC with a total volume of 2.0 mL (see Figures S6 and S7 in Section S4 for two photographs of the experimental setup). The FD cell for the unbounded configuration is a circular cell in which a gold electrode (diameter = 3.0 mm) has been inserted at the bottom, located below the BDD working electrode. This gold electrode is controlled potentiostatically for regenerating completely in a faradaic manner the 4CT^+^ moiety generated at the BDD working electrode which moves toward the gold electrode by linear diffusion, in a similar operation mode to the positive feedback mode of a Scanning Electrochemical Microscopy.^65^

The NPV potential waveform consists in the application of several constant potentials (of increasing amplitude) with identical time length τ. Between the application of two potentials of this sequence, the electrode is kept at a rest potential at which the regeneration of the faradaic converted species takes place. The time interval between two consecutive potentials at which *E*_rest_ is applied should be much greater under SID conditions, for the initial conditions to be restored.^3^ Since the charge transfer processes will be analyzed under FD conditions, the regeneration of the redox species will be complete in a relatively short time (2.0 s for the experimental conditions followed here), such that each individual potential of the complete sequence is equivalent to a single experiment with bulk concentrations of the species redox (see Figure S8 in Section S4 of the SI) and therefore, eq 7 can be used for calculating all the current–time transients of the potential waveform. Moreover, we have used long pulse times (τ ≥ 0.25 s) to avoid any possible resistive and capacitive influences, see Figures S9 and S10 in Section S5.

To verify the quasi-reversible nature of the oxidation of 4CT, Cyclic Voltammetry responses obtained under SID conditions for a solution of 4CT 2.08 mM at an aqueous 1.0 M KCl solution have been obtained with a BDD working electrode. These CV curves show peak-to-peak differences above 60 mV even for low values of the scan rate (see Figure S11 and Table S1 in Section S6 of the SI), a fact that clearly indicates that this process is far from being of reversible nature. By applying the Nicholson’s method to these responses,^1,2^ the following rate constant value (assuming a symmetric BV formalism, α = 0.5) is obtained: *k* ^0′^ = 2.9 × 10^–3^ cm s^–1^.

The diffusion coefficient has been obtained from a chronoamperometric analysis under limiting current conditions (*E*_applied_ = 1.30 V). From a Cottrell’s logarithmic analysis of the current–time transients (see Figure S12 in Section S5 of the SI), we have obtained a value *D*_4CT_ = 2.24 × 10^–6^ cm^2^ s^–1^, which is consistent with values previously reported *D*_4CT_ = 2.64 × 10^–6^ cm^2^ s^–1^.^64^

In order to determine the value of the rate constant for FD conditions, two different sets of NPV have been carried out under bounded and unbounded conditions. First, different pulse times for the NPV in the range 0.25–2.0 s have been assayed at a distance approximately constant (see Figure 7). Second, the NPV pulse time has been fixed (τ = 0.5 s) and different thincknesses for the diffusion field thickness have been assayed (see Figure 8).

**Figure 7:**
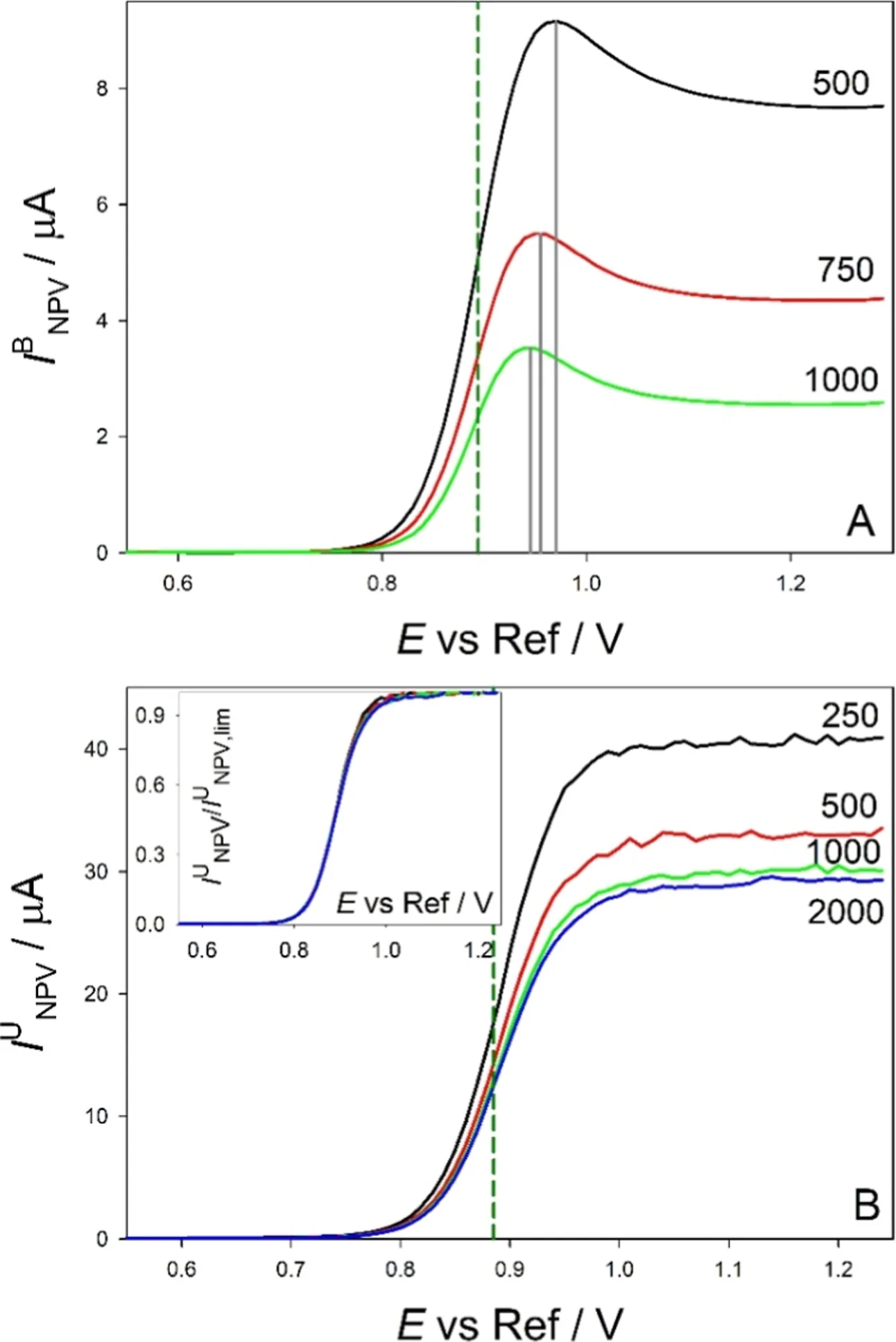
NPV responses for 4CT 2.085 mM + KCl 1.0 M in a BDD electrode for bounded (A) and unbounded (B) configurations. Different pulse time have been used, with the value in milliseconds indicated in the curves. *E*_rest_ = 0.400 V, *t*_rest_ = 2.0 s. The values of the operating distance for each curve are (A) τ = 0.5 s, *L* = 11.8 μm; τ = 0.75 s, *L* = 13.0 μm; τ = 1.0 s, *L* = 13.7 μm; (B) τ = 0.25 s, *L* = 15.2 μm; τ = 0.5 s, *L* = 18.4 μm; τ = 1.0 s, *L* = 20.0 μm; τ = 2.0 s, *L* = 20.6 μm; *T* = 298 K. Vertical green dashed line marks the position of the formal potential *E* ^0′^ = 0.894 V for (A) and *E* ^0′^ = 0.885 V for (B). *T* = 298 K. Inner (B) shows the normalized $I_{NPV}^{U}/I_{NPV,\lim}^{U}$ –*E* curves obtained from (B).

**Figure 8:**
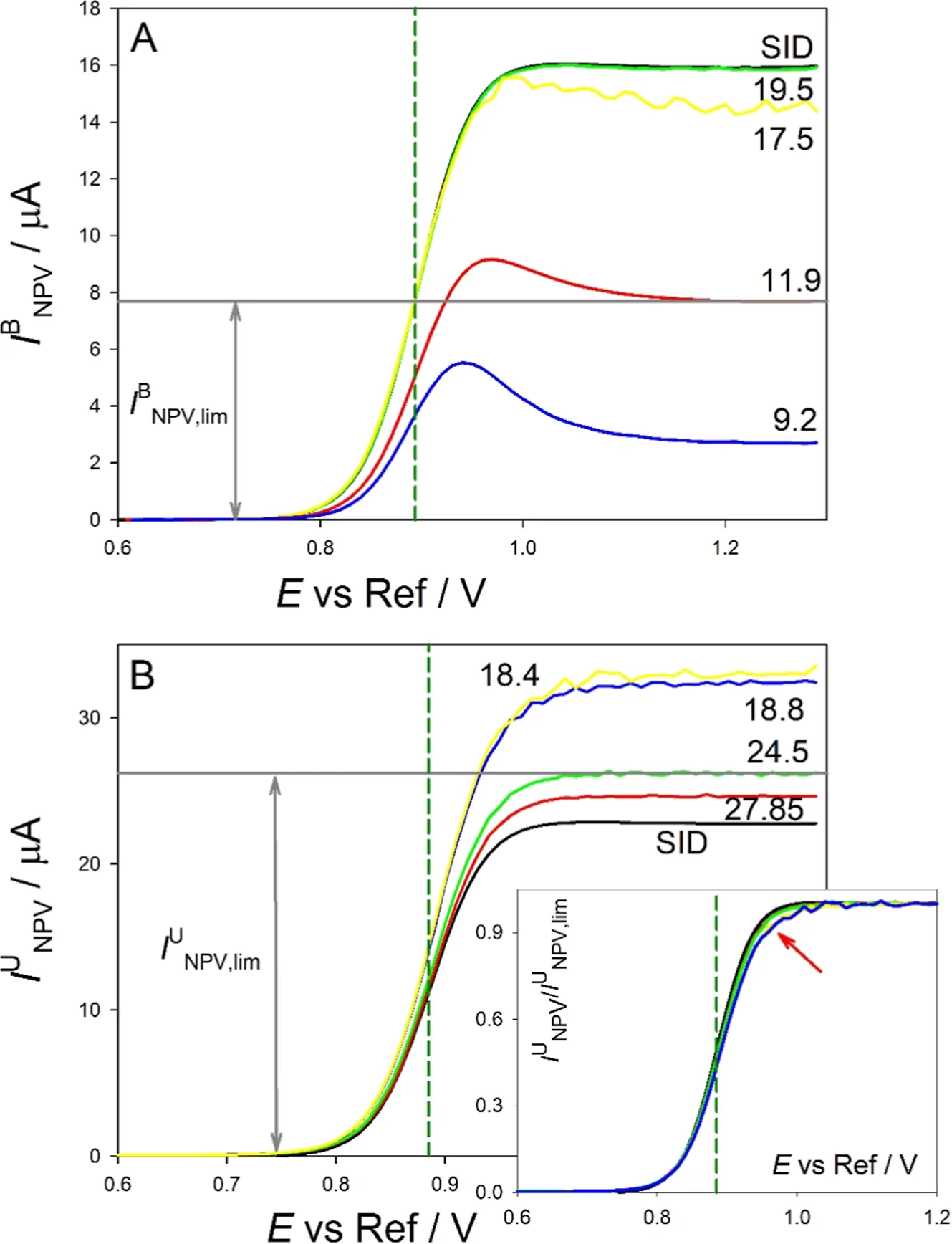
NPV responses for 4CT 2.085 mM + KCl 1.0 M in a BDD electrode for bounded (A) and unbounded (B) configurations. Different thickness of the diffusion field *L* has been used, with the value in microns, calculated by using eqs 25 and 26 for $I_{\lim}^{B}$ and $I_{\lim}^{U}$ , are indicated in the curves. *E*_rest_ = 0.400 V, *t*_rest_ = 2.0 s, τ = 0.5 s. *T* = 298 K. Vertical green dashed line marks the position of the formal potential *E* ^0′^ = 0.894 V for (A) and *E* ^0′^ = 0.885 V for (B). Inner (B) shows the normalized $I_{NPV}^{U}/I_{NPV,\lim}^{U}$ –*E* curves obtained from (B).

Figure 7 shows the effect of the NPV pulse time in the responses of 4CT for distances in the range (11.8–13.7) microns for the bounded case (Figure 7A) and (15–20.6) microns for the unbounded case (Figure 7B). Pulse times in the range 0.25–2.0 s have been used in such a way that the total duration of the complete NPV scans are in the range 190–270 s.

As can be seen in all the cases, the increase of τ leads to a decrease of the NPV currents for both bounded and unbounded cases (due to a lower sampled current value of the transient response). In both bounded and unbounded cases, constant currents are obtained at positive enough potentials (with small oscillations in the limiting currents obtained for the unbounded case for *L* ≤ 20 μm, with deviations from the medium value below 1.5%). As stated in the theoretical section, this limiting current is only dependent on the pulse time, the value of *L* and the diffusion coefficient, see eqs 25 and 26 and ref 28 Therefore, by using these Eqns. it is possible to determine the operational value of *L* for each NPV curve.

For the bounded curves in Figure 7A, a clear peak current is observed for all the pulse times considered. The increase of the pulse time from 0.5 to 1.0 s leads to an increase of the dimensionless rate constant ${\mathrm{k̅}}_{B,FD}^{0^{\prime }}$ through the term $\left(\sqrt{\tau /D}+\left(\tau /L\right)\right)$ (see eq 24). This increase affects both to the peak current, which decreases with the ${\mathrm{k̅}}_{B,FD}^{0^{\prime }}$ value (see Figure 5A), and to the peak potential, which moves toward the formal potential (vertical dashed green line in Figure 7). The half peak width is not affected by the pulse time in these conditions and it presents a value of 90 mV.

In the unbounded case shown in Figure 7B, the sigmoidal NPV curves also decrease as τ increases, but the dependence on the pulse time is progressively lost since, at long enough values, the response becomes stationary (see eq 20). Moreover, no significant shift of the NPV curves with the potential is observed, as shown by the normalized NPV curves in the inner Figure 7B, indicating a relatively small kinetic influence for the values of *L* and τ selected.

In agreement with eq 24 for the expressions of the dimensionless rate constants ${\mathrm{k̅}}_{B,FD}^{0^{\prime }}$ and ${\mathrm{k̅}}_{U,FD}^{0^{\prime }}$ , the increase of the pulse time affects in a different way to the TL limits of the kinetic behavior of the bounded and unbounded responses ( ${\mathrm{k̅}}_{B,FD}^{0^{\prime }}={\mathrm{k̅}}_{SID}^{0^{\prime }}+{\mathrm{k̅}}_{B,TL}^{0^{\prime }}=k^{0^{\prime }}\left(\sqrt{\tau /D}+\left(\tau /L\right)\right)$ and ${\left({\overset{¯}{k}}_{U,FD}^{0^{\prime }}\right)}^{-1}={\left({\overset{¯}{k}}_{SID}^{0^{\prime }}\right)}^{-1}+{\left({\overset{¯}{k}}_{U,T\,\;L}^{0^{\prime }}\right)}^{-1}=\sqrt{D/\tau }/k^{0^{\prime }}+D/\left(k^{0^{\prime }}L\right)$ ). For this reason, and in order to isolate the effect of finite diffusion on the overall kinetic influence, the NPV responses corresponding to variations in the length *L* of the diffusion field have been analyzed, while keeping the pulse duration constant (so that only the ${\mathrm{k̅}}_{B,TL}^{0^{\prime }}$ and ${\mathrm{k̅}}_{U,TL}^{0^{\prime }}$ terms of the dimensionless constants are affected).

Thus, Figure 8 shows the effect of *L* on the NPV curves obtained under bounded and unbounded conditions for the same time pulse (τ = 0.5 s, and a total time for the complete NPV scan of 225 s). In an opposite way to that observed for the influence of τ discussed above, the narrowing of the diffusion field leads to smaller currents for the bounded configuration (and to a decrease of the kinetic influence), whereas the current increases for the unbounded case (and the influence of kinetics becomes also more relevant). The values of *L* for each NPV curves have been calculated from the values of the limiting currents at positive potentials, $I_{\lim}^{B}$ and $I_{\lim}^{U}$ , by using eqs 25 and 26, for the fixed value of pulse time and the diffusion coefficient of 4CT previously obtained. In Figure 8A,B, the values of this limiting currents for *L* = 11.9 and 24.5 μm, respectively, have been indicated with gray lines. For values of *L* ≤ 20 μm, small oscillations are observed in the limiting current values for the unbounded case, with deviations from the medium value below 0.5% (see also Figure 7B), Thus, for the bounded case, the decrease of *L* leads to lower NPV currents and to a change in the shape of the responses, from sigmoidal curves for the higher values of the distance to the appearance of a clear peak current for the smaller *L*. In the curves of Figure 8A, an 84% reduction is observed in the limiting current when the distance decreases by 10 μm. As in the NPV responses of Figure 7A, the peak position for the smaller distances moves toward the formal potential. Both features are a consequence of the increase of the overall dimensionless rate constant ${\mathrm{k̅}}_{B,FD}^{0^{\prime }}$ through an increase of the TL term ${\mathrm{k̅}}_{B,TL}^{0^{\prime }}=k^{0^{\prime }}\tau /L$ (see eq 23), typical of the behavior of a quasi-reversible process under FD conditions, as shown in Figure 2.

On the opposite way, for the unbounded case (Figure 8B), the current increases as *L* decreases, with the NPV curves in this Figure showing an increase of 1.5 times when moves from SID conditions (black line) to 18.4 μm (yellow line). The NPV curves are slightly shifted toward more positive potentials although this displacement is small (but it can be clearly seen, especially at the potential region close to the limiting current, as shown in the Inner Figure 8B, see red arrow).

In order to determine the kinetic parameters of the process, a fitting procedure focused on the NPV responses has been followed (see Section S7 and Figure S13 for a detailed description of the protocol followed). Equations 7–12 have been used, with the rate constant, *k* ^0′^ and α (BV), or *k* ^0′^ and γ (MHC) as unknowns to be determined. We have assumed a value of λ = 1 eV for the MHC formalism, which is typical for oxidation processes of organic molecules.^51^ For the formal potential, a seed value equal to the semisum of the SID CV peak potentials have been used (*E* ^0′^ = 0.894 V) although this value is only an estimative one since we have no prior knowledge about the symmetry of the kinetic formalism. Values for the diffusion coefficient are *D*_4CT_ = (2.24–4.25) × 10^–6^ cm^2^ s^–1^, obtained from Chronoamperometry under SID conditions.

We have analyzed NPV curves obtained for the smallest values of *L* under bounded (for which a clear peak is observed) and unbounded conditions, and different sets of values of *k* ^0′^ and α (BV), or *k* ^0′^ and γ (MHC) have been tested.

The parameters selected, given in Table 1, have been obtained by combining the results with a higher degree of agreement obtained for both bounded and unbounded conditions. Small differences in the formal potential values (around 9 mV) previously estimated from the CV curves (see Section S6 in SI) have been obtained.

**Table 1:** Kinetic, Thermodynamic and Mass Transport Parameters for the Oxidation of 4CT at a BDD Electrode Obtained From the Fittings of Experimental Responses in Figures 7 and 8 with Theoretical NPV Curves Calculated by Using Eq 7

configuration	*L*/μm	*E* ^0′^/V	*k* ^0′^/cm s^–1^	1 – α	γ	*D*_4CT_/cm^2^ s^–1^
bounded	13.7	0.894	3.7 × 10^–3^	0.57	–0.4	2.24 × 10^–6^
unbounded	20.0	0.885	5.0 × 10^–3^	0.70	–0.4	4.25 × 10^–6^

By using eq 7 with the parameters shown in Table 1 we have obtained different sets of theoretical NPV curves for both B and U conditions. A comparison between experimental and theoretical responses is shown in Figure 9 for a potential pulse time of 1.0 s. As can be seen in this figure, the degree of coincidence is significant, especially for the MHC formalism in the unbounded configuration (see solid black line and dashed blue line in Figure 9B), indicating a higher ability of this formalism for accurately describe the quasi-reversible responses of 4CT.

**Figure 9:**
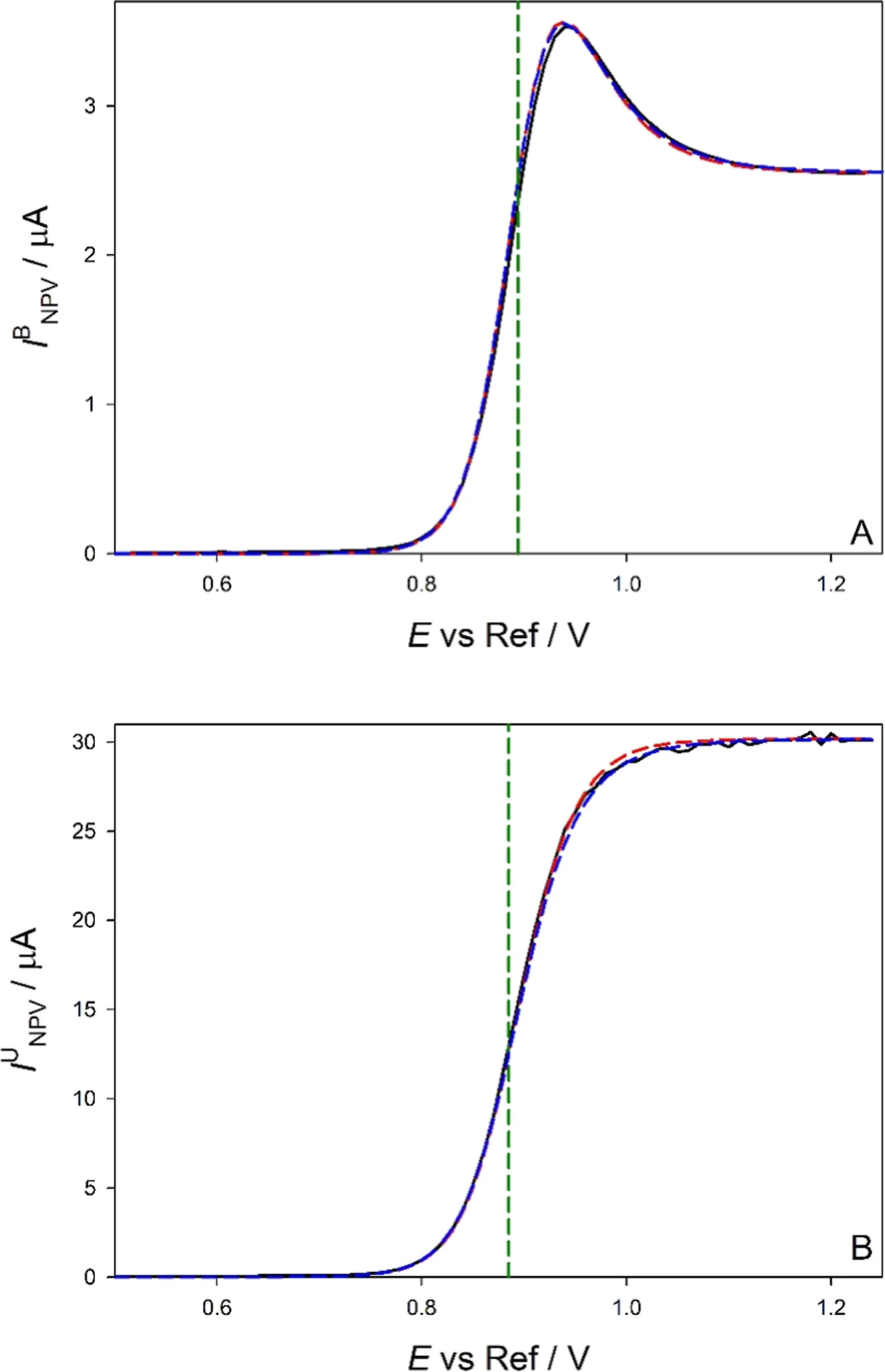
Solid black lines: experimental NPV curves for 4CT 2.085 mM + KCl 1.0 M in a BDD electrode for bounded (A) and unbounded (B) configurations. τ = 1.0 s. The values of *L* (in microns) are 13.7 (A) and 20.0 (B). *T* = 298 K. Dashed lines: theoretical NPV curves calculated by using eq 7 with the parameters given in Table 1—red (BV), blue (MHC). Vertical dashed green lines mark the fitted values for the formal potential *E* ^0′^ = 0.894 V for (A) and *E* ^0′^ = 0.885 V for (B).

In agreement with Table 1 and Figure 9, the oxidation of 4CT is a quasi-reversible process (the rate constant is in the range (3.7–5.0) × 10^–3^ cm s^–1^). Moreover, this is an asymmetric charge transfer reaction in terms of the anodic charge transfer coefficient (BV) or the asymmetry parameter (MHC). Note that the MHC formalism presents a higher degree of coherence in the symmetry parameter (for BV, (1 – α) has differences for both B and U configurations and shows very high values, especially in the U case, whereas for MHC the parameter γ has the same value), and in the case of the unbounded configuration, for which the redox kinetics influence is higher, the MHC curve reproduces in a more accurate way the experimental NPV response (see the upper region of the unbounded curves in Figure 9B).

From the parameters in Table 1 and the values of the NPV pulse time and the thickness of the diffusion field it is possible to calculate the values of the dimensionless rate constants for both bounded and unbounded conditions by using eq 24. For the bounded curves in Figure 9A, the SID value is ${\mathrm{k̅}}_{SID}^{0^{\prime }}=3.34$ , whereas for *L* = 13.7 μm, the dimensionless rate constant is ${\mathrm{k̅}}_{FD}^{0^{\prime }}=6.98$ , that is to say, it doubles in value compared with the SID limit (see Figure 2). However, for the unbounded case, the opposite behavior is obtained. The SID limit is ${\mathrm{k̅}}_{SID}^{0^{\prime }}=2.42$ and the dimensionless rate constant for *L* = 20 μm is ${\mathrm{k̅}}_{FD}^{0^{\prime }}=1.20$ , i. e., the constant is set to half the SID limit value (see Figure 2).

We can therefore conclude that NPV technique enables a quantitative analysis of the influence of the mass transport mechanism on the response of quasi-reversible and irreversible charge transfer processes, revealing marked differences between the conditions of depletion (B) and regeneration (U) of the redox probe.

## Conclusions

Normal pulse voltammetry has been revealed to be a simple and highly effective technique for characterizing quasi-reversible charge transfer processes under Finite Diffusion (FD) conditions. The current–potential-time expression given here (eq 7) enables the analysis of the response of these processes under different experimental conditions with great accuracy.

Significantly, the dominant mass transport mechanism gives rise to differences in the kinetic influences on the overall response. When diffusive depletion of the electroactive species occurs (bounded conditions), spatial confinement makes the response more reversible than under Semi-Infinite Diffusion (SID) conditions. This results in a current peak appearing in the NPV response, enabling the charge transfer constant values to be determined accurately. Conversely, when the redox probe regenerates under FD conditions (unbounded conditions), the irreversibility of the response increases with decreasing solution layer thickness, resulting in a dimensionless kinetic constant ( ${\mathrm{k̅}}_{U,TL}^{0^{\prime }}=k^{0^{\prime }}\left(L/D\right)$ ) that is formally identical to that obtained for ultramicroelectrodes under radial diffusion conditions (see, for example, refs 3 and 56 ). This is because, under Thin-Layer (TL) conditions and for a bounded configuration, the influence of the diffusion of electroactive species on the response vanishes (i.e., the current is analogous to that obtained for a diffusionless system^57^), whereas for an unbounded configuration, the regeneration effect enhances mass transport (as in the case of microelectrodes), making it very fast despite being linear diffusion.

From a practical point of view, it should be noted that most of the protocols reported in the literature for determining kinetic parameters are based on two configurations: a SID field (Nicholson method), or application of the Koutecky–Levich equation to the response of a process using a rotating disc electrode.^38^ These are the reference methodologies for carrying out kinetic analysis of redox-active species with potential applications in redox flow batteries. However, these methodologies contrast with the fact that the redox conversion of these species takes place under conditions of spatial confinement, in which kinetic influences can vary significantly depending on the architecture of the devices used, as demonstrated in the present study. This conclusion therefore serves as a wake-up call to researchers in the field, urging them to recognize the crucial importance of mass transport in kinetic studies, as this can compromise the efficacy of electrochemical devices operating under spatial confinement.

The Butler–Volmer (BV) and Marcus–Hush–Chidsey (MHC) formalisms were used to characterize the response. While both approaches lead to good agreement between the theoretical and experimental results (although with high values of the charge transfer coefficient in the BV case), MHC appears to offer a higher degree of coherence in the obtained kinetic parameters and reproduces the responses with greater accuracy.

The results obtained demonstrate the validity and accuracy of the NPV technique. In the case of the oxidation of the 4-carboxy-TEMPO radical, a complete characterization of the redox response under both bounded and unbounded configurations enables the opposite influences of bounded and unbounded mass transport on the overall kinetic behavior to be identified and indicates the superiority of the MHC formalism for adequately comprehending the charge transfer processes.

Finally, we can conclude that there is a clear and urgent need for new theoretical frameworks developed specifically for these new experimental scenarios, in order to provide simple and accurate tools that enable us to characterize the kinetics at play and correctly elucidate the reaction mechanisms involved. The NPV technique described in this paper represents a first step in this direction, as it adapts existing theory to these new relevant domains.

## Supplementary Material



## Notes

Expression of the rate constants for oxidation and reduction processes for Butler–Volmer and Marcus–Hush–Chidsey kinetic formalisms; theoretical deduction of the expression of the current; additional theoretical results; experimental conditions; chronoamperometric analysis for bounded conditions; fitting protocol; additional experimental results (PDF)
