# Supplementary material for: Measuring Electrode Kinetics Under Spatial Confinement. Application of Pulse Voltammetry to Different Mass Transport Modes Using Butler–Volmer and Marcus–Hush–Chidsey Frameworks
Source: ACS Meas Sci Au. 2026 May 26;6(4):1239–55. doi: 10.1021/acsmeasuresciau.6c00096 (PMC13495712; doi:10.1021/acsmeasuresciau.6c00096)
Supplement: Supplementary file 1 [file tg6c00096_si_001.pdf]

Supporting Information of manuscript
Measuring electrode kinetics under spatial confinement. Application of pulse voltammetry to different mass transport modes using Butler-Volmer and Marcus-Hush-Chidsey frameworks

José V. Hernández-Tovar, Antonio J. Martínez-García, Francisco Martínez-Ortiz, Eduardo Laborda, Manuela López-Tenés, Joaquín González*

Departamento de Química Física, Facultad de Química, Regional Campus of International Excellence "Campus Mare Nostrum", Universidad de Murcia, 30100 Murcia, Spain

***corresponding author e-mail: josquin@um.es**

S1. Expressions of the rate constants for oxidation and reduction processes for Butler-Volmer and Marcus-Hush-Chidsey kinetic formalisms.
S2. Theoretical deduction of the expression of the current
S3. Additional theoretical results
S4. Experimental conditions.
S5. Chronoamperometric analysis for bounded conditions
S6. Additional experimental results
S7. Fitting protocol
S8. References

S1. Expressions of the rate constants for oxidation and reduction processes for Butler-Volmer and Marcus-Hush-Chidsey kinetic formalisms.

Let us consider the following heterogeneous slow charge transfer process, taking place at the interface between an electrode and an electrolytic solution due to the application of a constant potential E,

$$\mathrm{R} \underset{k_{\mathrm{red}}}{\overset{k_{\mathrm{ox}}}{\rightleftarrows}} \mathrm{O} + \mathrm{e}^{-} \qquad \text{(I)}$$

with R and O being reduced and oxidised species at initial concentrations c_{R}^{*} and c_{O}^{*}, respectively. k_{ox} and k_{red} are the potential dependent rate constants for the oxidation and reduction processes, respectively.

The expression for the rate constant k_{red} depends on the kinetic formalism, and two of them will be considered here, the Butler-Volmer (BV) and the asymmetric Marcus-Hush-Chidsey (MHC) one, for which k_{red} is given as (see [S1-S5])

$$k_{\mathrm{red}} = k^{0'} g(\eta) \text{ with } g(\eta) = \begin{cases} g^{\mathrm{BV}}(\eta) = \mathrm{e}^{-\alpha\eta} \\ g^{\mathrm{MHC}}(\eta) = \dfrac{F(\eta,\Lambda)}{F(0,\Lambda)} = \dfrac{\displaystyle\int_{-\infty}^{\infty} \left(\dfrac{\mathrm{e}^{-\Delta G^{\neq}_{\mathrm{red}}(\varepsilon,\eta,\Lambda,\gamma)}}{1+\mathrm{e}^{-\varepsilon}} \right) d\varepsilon}{\displaystyle\int_{-\infty}^{\infty} \left(\dfrac{\mathrm{e}^{-\Delta G^{\neq}_{\mathrm{red}}(\varepsilon,0,\Lambda,\gamma)}}{1+\mathrm{e}^{-\varepsilon}} \right) d\varepsilon} \end{cases} \qquad \text{(S1)}$$

where

$$\eta = \frac{F}{RT}\left(E - E^{0'}\right) \qquad \text{(S2)}$$

In Eqns. (S1)-(S2), $k^{0'}$ is the conditional rate constant, α is the charge transfer coefficient, $E^{0'}$ is the formal potential and

$$\Delta G^{\neq}_{\mathrm{red}}(\varepsilon,\eta,\Lambda,\gamma) = \frac{\Lambda}{4}\left(1+\frac{\eta+\varepsilon}{\Lambda}\right) + \gamma\left(\frac{\eta+\varepsilon}{4}\right)\left(1+\left(\frac{\eta+\varepsilon}{\Lambda}\right)^{2}\right) + \gamma\frac{\Lambda}{16} \qquad \text{(S3)}$$

Λ is the dimensionless reorganization energy given by

$$\Lambda = \frac{\lambda}{RT} \qquad \text{(S4)}$$

where λ is the molar reorganization energy [S4, S6, S7], and γ is the asymmetry parameter that accounts for differences between the inner-shell force constants of the oxidized and the reduced species (a positive γ value is associated with greater force constants for the oxidized species and the opposite for negative γ values). The case $\gamma = 0$ (equal force constants for both reduced and oxidized states) corresponds to the simpler symmetrical Marcus-Hush-Chidsey model. Note that for both MHC and BV kinetics formalisms is fulfilled that

$$\frac{k_{ox}}{k_{red}} = e^{\eta} \quad \text{(S5)}$$

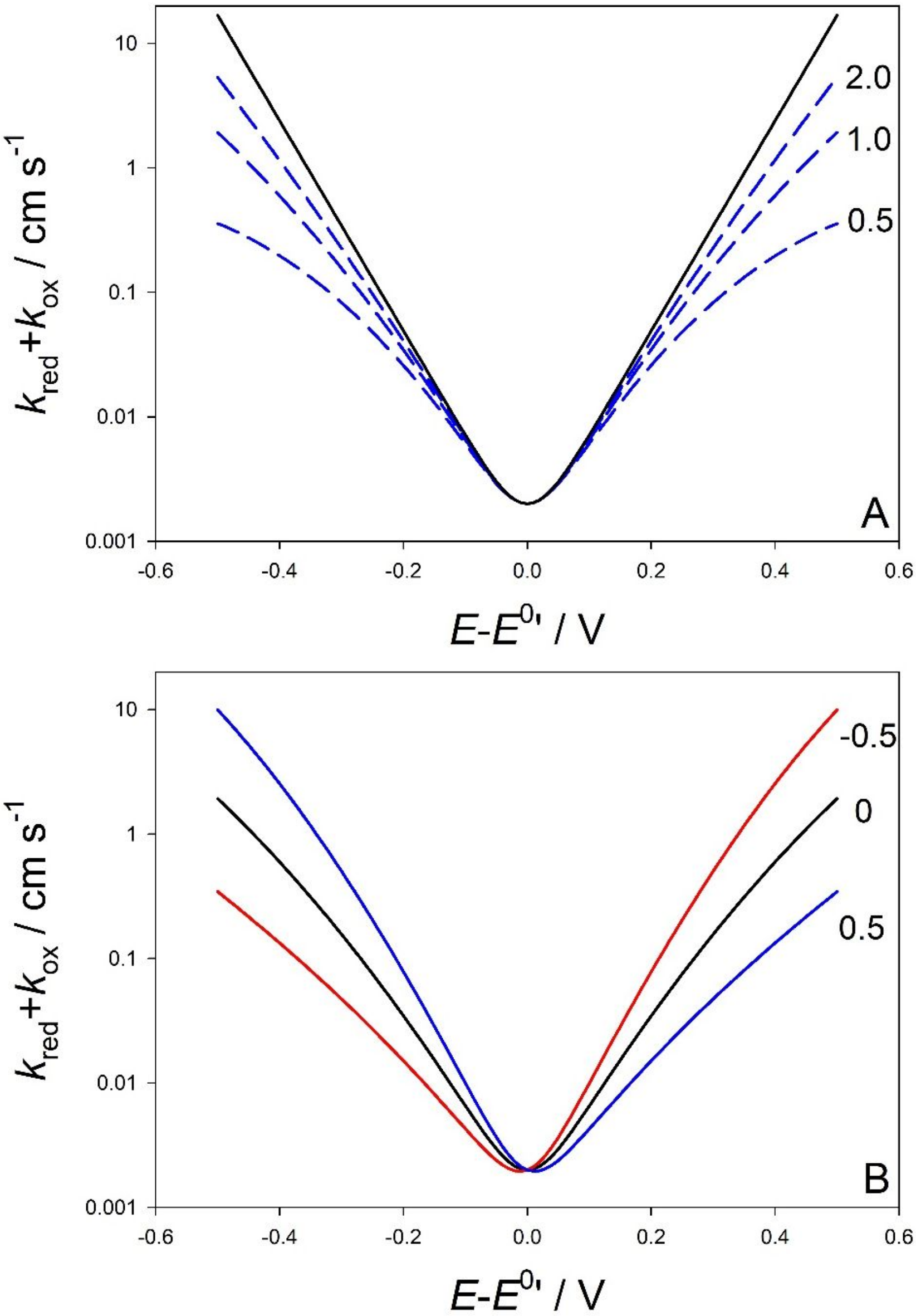

Figure S1. the evolution of the sum $(k_{red} + k_{ox})$ with the difference $\left(E - E^{0'}\right)$ calculated from Eqns. (S1)-(S5). S1A. Influence of λ (values in eV shown in the curves) for symmetrical MHC model ($\gamma = 0$, dashed blue lines), compared to a symmetrical BV approach ($\alpha = 0.5$, solid black lines). S1B. Effect of the asymmetry of the charge transfer reaction through parameter γ (values in the curves) for a MHC formalism when $\lambda = 1.0$ eV. $k^{0'} = 10^{-3}$ cm s^{-1}, T=298 K.

Figure S1 shows the evolution of the sum $(k_{red} + k_{ox})$ with the difference $E - E^{0'}$ in terms of λ (Fig. S1A, values in eV shown in the curves) for symmetrical MHC (dashed blue lines), compared to a symmetrical BV approach (solid black lines), and the influence of the

asymmetry of the charge transfer reaction through parameter γ (values in the curves) for a MHC formalism when $\lambda = 1.0$ eV.

S2. Theoretical deduction of the expression of the current

By assuming that the diffusion coefficients of both electroactive species are equal, i.e., $D_O = D_R = D$, and that the mass transport is only by linear diffusion, the following differential equation describes the time and spatial variation of species O and R for the charge transfer process given by Scheme S1 (second Fick's law), when a constant potential is applied,

$$\frac{\partial c_i^{B/U}}{\partial t} = D\left(\frac{\partial^2 c_i^{B/U}}{\partial x^2}\right) \qquad \text{i=O, R} \tag{S6}$$

which can be re-written in terms of the following new variables

$$\left.\begin{aligned} \tau &= \frac{D}{L^2}t \\ \xi &= \frac{x}{L} \end{aligned}\right\} \tag{S7}$$

as

$$\frac{\partial c_i^{B/U}}{\partial \tau} = \left(\frac{\partial^2 c_i^{B/U}}{\partial \xi^2}\right) \qquad \text{i=O, R} \tag{S8}$$

Superscripts "B" and "U" refer to the bounded and unbounded mass transfer modes, respectively.

This differential equation system has the following boundary value problem:

For $\tau = 0,\ 0 \le \xi \le 1$,

$$c_i^{B/U}(\xi, \tau) = c_i^* \qquad \text{i=O, R} \tag{S9}$$

For $\tau > 0,\ \xi = 0$,

$$\left(\frac{\partial c_O^{B/U}}{\partial \xi}\right)_{\xi=0} = -\left(\frac{\partial c_R^{B/U}}{\partial \xi}\right)_{\xi=0} \tag{S10}$$

and since the charge transfer process shows a finite kinetics,

$$\frac{I^{B/U}}{FA}\frac{L}{D} = \tilde{k}_{ox} c_R^s - \tilde{k}_{red} c_O^s \tag{S11}$$

with c_i^s (i=O,R) being the surface concentrations (at $\xi = 0$) of species i (whose value is independent of the mass transport mode), and

$$\left.\begin{aligned} \tilde{k}_{ox} &= k_{ox}\frac{L}{D} \\ \tilde{k}_{red} &= k_{red}\frac{L}{D} \end{aligned}\right\} \tag{S12}$$

The expressions of the potential dependence of both rate constants is given by Eqns. (S1)-(S5) for both BV and MHC formalisms.

For bounded conditions it is fulfilled that for $\tau > 0,\ \xi = 1$,

$$\left(\frac{\partial c_{\mathrm{O}}^{\mathrm{B}}}{\partial \xi}\right)_{\xi=1} = \left(\frac{\partial c_{\mathrm{R}}^{\mathrm{B}}}{\partial \xi}\right)_{\xi=1} = 0 \qquad \text{Bounded} \qquad \text{(S13)}$$

whereas for unbounded conditions it is fulfilled that for $\tau > 0,\ \xi = 1$,

$$c_{\mathrm{i}}^{\mathrm{U}}\left(\xi = 1, \tau\right) = c_{\mathrm{i}}^{*} \qquad \mathrm{i=O,\ R} \qquad \text{(S14)}$$

The current for both configurations is given by

$$\frac{I^{\mathrm{B/U}}}{FA}\frac{L}{D} = \left(\frac{\partial c_{\mathrm{R}}^{\mathrm{B/U}}}{\partial \xi}\right)_{\xi=0} = -\left(\frac{\partial c_{\mathrm{O}}^{\mathrm{B/U}}}{\partial \xi}\right)_{\xi=0} \qquad \text{(S15)}$$

By applying the Laplace's transform to Eqns. (S8)-(S14) it is obtained,

$$\frac{d^2\bar{c}_{\mathrm{i}}^{\mathrm{B/U}}}{d\xi^2} - p\bar{c}_{\mathrm{i}}^{\mathrm{B/U}} = -c_{\mathrm{i}}^{*} \qquad \mathrm{i=O,\ R} \qquad \text{(S16)}$$

where $\bar{c}_{\mathrm{i}}^{\mathrm{B/U}}\left(\xi, p\right)$ denotes the expressions of the concentration profile of species i in the Laplace space.

$$\left(\frac{d\bar{c}_{\mathrm{O}}^{\mathrm{B/U}}}{d\xi}\right)_{\xi=0} = -\left(\frac{d\bar{c}_{\mathrm{R}}^{\mathrm{B/U}}}{d\xi}\right)_{\xi=0} \qquad \text{(S17)}$$

For bounded conditions it is fulfilled that

$$\left(\frac{d\bar{c}_{\mathrm{O}}^{\mathrm{B}}}{d\xi}\right)_{\xi=1} = \left(\frac{d\bar{c}_{\mathrm{R}}^{\mathrm{B}}}{d\xi}\right)_{\xi=1} \qquad \text{(S18)}$$

whereas for unbounded conditions,

$$\bar{c}_{\mathrm{i}}^{\mathrm{U}}\left(\xi = 1\right) = \frac{c_{\mathrm{i}}^{*}}{p} \qquad \mathrm{i=O,\ R} \qquad \text{(S19)}$$

The Laplace's transform of the current is given by (see Eqn. (S15)),

$$\frac{\bar{I}^{\mathrm{B/U}}}{FA}\frac{L}{D} = \left(\frac{d\bar{c}_{\mathrm{R}}^{\mathrm{B/U}}}{d\xi}\right)_{\xi=0} = -\left(\frac{d\bar{c}_{\mathrm{O}}^{\mathrm{B/U}}}{d\xi}\right)_{\xi=0} = \tilde{k}_{\mathrm{ox}}\bar{c}_{\mathrm{R}}^{\mathrm{s,\ B/U}} - \tilde{k}_{\mathrm{red}}\bar{c}_{\mathrm{O}}^{\mathrm{s,B/U}} \qquad \text{(S20)}$$

The solution to differential equation (S16) is the following,

$$\bar{c}_{\mathrm{i}}^{\mathrm{B/U}} = \frac{c_{\mathrm{i}}^{*}}{p} + A_{\mathrm{i}}^{\mathrm{B/U}}e^{-\sqrt{p}\xi} + B_{\mathrm{i}}^{\mathrm{B/U}}e^{\sqrt{p}\xi} \qquad \mathrm{i=O,\ R} \qquad \text{(S21)}$$

where $A_{\mathrm{i}}^{\mathrm{B/U}}$ and $B_{\mathrm{i}}^{\mathrm{B/U}}$ will be obtained from the boundary value problem. The derivative of $\bar{c}_{\mathrm{i}}^{\mathrm{B/U}}$ at $\xi = 0$ is given by,

$$\left(\frac{d\overline{c}_{\mathrm{i}}^{\mathrm{B/U}}}{d\xi}\right)_{\xi=0} = \sqrt{p}\left(-A_{\mathrm{i}}^{\mathrm{B/U}} + B_{\mathrm{i}}^{\mathrm{B/U}}\right) \qquad \mathrm{i=O,\,R} \tag{S22}$$

Therefore, the current is given by (see Eqn. (S20)),

$$\frac{\overline{I}^{\mathrm{B/U}}}{FA}\frac{L}{D} = \sqrt{p}\left(-A_{\mathrm{R}}^{\mathrm{B/U}} + B_{\mathrm{R}}^{\mathrm{B/U}}\right) = -\sqrt{p}\left(-A_{\mathrm{O}}^{\mathrm{B/U}} + B_{\mathrm{O}}^{\mathrm{B/U}}\right) \tag{S23}$$

By applying condition (S17) to the solution given by (S21) it is obtained,

$$-A_{\mathrm{R}}^{\mathrm{B/U}} + B_{\mathrm{R}}^{\mathrm{B/U}} = A_{\mathrm{O}}^{\mathrm{B/U}} - B_{\mathrm{O}}^{\mathrm{B/U}} \tag{S24}$$

For bounded conditions at $\xi = 1$, it is obtained (see Eqn. (S18)):

$$-A_{\mathrm{R}}^{\mathrm{B}} e^{-\sqrt{p}} + B_{\mathrm{R}}^{\mathrm{B}} e^{\sqrt{p}} = -A_{\mathrm{O}}^{\mathrm{B}} e^{-\sqrt{p}} + B_{\mathrm{O}}^{\mathrm{B}} e^{\sqrt{p}} = 0 \tag{S25}$$

whereas for unbounded conditions at $\xi = 1$, it is deduced (see Eqn. (S19)),

$$A_{\mathrm{R}}^{\mathrm{U}} e^{-\sqrt{p}} + B_{\mathrm{R}}^{\mathrm{U}} e^{\sqrt{p}} = A_{\mathrm{O}}^{\mathrm{U}} e^{-\sqrt{p}} + B_{\mathrm{O}}^{\mathrm{U}} e^{\sqrt{p}} = 0 \tag{S26}$$

By combining Eqns. (S23)-(S25) it is possible to obtain an expression of coefficients $A_{\mathrm{i}}^{\mathrm{B}}$ and $B_{\mathrm{i}}^{\mathrm{B}}$ in terms of the current,

$$\begin{aligned} B_{\mathrm{O}}^{\mathrm{B}} &= -B_{\mathrm{R}}^{\mathrm{B}} \\ A_{\mathrm{O}}^{\mathrm{B}} &= -A_{\mathrm{R}}^{\mathrm{B}} \end{aligned} \tag{S27}$$

In the same way, from Eqns. (S23), (S24) and (S26), the following expressions for $A_{\mathrm{i}}^{\mathrm{U}}$ and $B_{\mathrm{i}}^{\mathrm{U}}$ are obtained,

$$\begin{aligned} B_{\mathrm{O}}^{\mathrm{U}} &= -B_{\mathrm{R}}^{\mathrm{U}} \\ A_{\mathrm{O}}^{\mathrm{U}} &= -A_{\mathrm{R}}^{\mathrm{U}} \end{aligned} \tag{S28}$$

By combining Eqns. (S23) for the current and (S25)-(S28) it is obtained,

$$\begin{aligned} \frac{\overline{I}^{\mathrm{B}}}{FA}\frac{L}{D} &= -\sqrt{p}\,B_{\mathrm{R}}^{\mathrm{B}}\left(1 - e^{2\sqrt{p}}\right) \\ \frac{\overline{I}^{\mathrm{U}}}{FA}\frac{L}{D} &= \sqrt{p}\,B_{\mathrm{R}}^{\mathrm{U}}\left(1 + e^{2\sqrt{p}}\right) \end{aligned} \tag{S29}$$

The expressions of coefficients $B_{\mathrm{R}}^{\mathrm{B}}$ and $B_{\mathrm{R}}^{\mathrm{U}}$ can be obtained by combining Eqns. (S20)-(S28),

$$\begin{aligned} B_{\mathrm{R}}^{\mathrm{B}} &= \frac{\tilde{k}_{\mathrm{ox}} c_{\mathrm{R}}^{*} - \tilde{k}_{\mathrm{red}} c_{\mathrm{O}}^{*}}{p} \frac{1}{\sqrt{p}\left(1 - e^{2\sqrt{p}}\right) + \tilde{k}_{\mathrm{T}}\left(1 + e^{2\sqrt{p}}\right)} \\ B_{\mathrm{R}}^{\mathrm{U}} &= \frac{\tilde{k}_{\mathrm{ox}} c_{\mathrm{R}}^{*} - \tilde{k}_{\mathrm{red}} c_{\mathrm{O}}^{*}}{p} \frac{1}{\sqrt{p}\left(1 + e^{2\sqrt{p}}\right) + \tilde{k}_{\mathrm{T}}\left(1 - e^{2\sqrt{p}}\right)} \end{aligned} \tag{S30}$$

By inserting Eqns. (S30) into (S28) the following expression for the current is immediately deduced:

$$\frac{\bar{I}^{\mathrm{B/U}}}{FA}\frac{L}{D}=\frac{\tilde{k}_{\mathrm{ox}}c_{\mathrm{R}}^{*}-\tilde{k}_{\mathrm{red}}c_{\mathrm{O}}^{*}}{\sqrt{p}}\frac{1}{\sqrt{p}+\tilde{k}_{\mathrm{T}}f^{\mathrm{B/U}}(p)} \tag{S31}$$

where

$$\begin{aligned} f^{\mathrm{B}}(p) &= \coth\left(\sqrt{p}\right) \\ f^{\mathrm{U}}(p) &= \tanh\left(\sqrt{p}\right) \end{aligned} \tag{S32}$$

a. Limiting cases for the current

From the general expression for the current given by Eqn. (S31) it is possible to deduce particular cases in terms of the values of parameter p. Although this parameter only has sense in the Laplace space, it relates to the ratio between the thickness of the solution and the linear diffusion layer, $L/\sqrt{Dt}$. Thus, SID conditions, $L >> \sqrt{Dt}$, are equivalent to the limit $p \to \infty$, whereas for TL conditions, $L << \sqrt{Dt}$, the limit $p \to 0$ applies.

a.1. Semi-Infinite Diffusion (SID) Limit

By imposing the condition $p \to \infty$ into equation (S31), and taking into account that $f^{\mathrm{B/U}}(p \to \infty) = 1$ [S8], it is obtained,

$$\frac{\bar{I}^{\mathrm{SID}}}{FA}\frac{L}{D}=\frac{\tilde{k}_{\mathrm{ox}}c_{\mathrm{R}}^{*}-\tilde{k}_{\mathrm{red}}c_{\mathrm{O}}^{*}}{\sqrt{p}}\frac{1}{\sqrt{p}+\tilde{k}_{\mathrm{T}}} \tag{S33}$$

By carrying out the inverse of the Laplace transform of Eqn. (S33), and taking into account Eqns. (S12) it can be deduced the following expression for the current,

$$I^{\mathrm{SID}}=FA\left(k_{\mathrm{ox}}c_{\mathrm{R}}^{*}-k_{\mathrm{red}}c_{\mathrm{O}}^{*}\right)e^{\left(k_{\mathrm{T}}^{2}\frac{t}{D}\right)}erfc\left(k_{\mathrm{T}}\sqrt{\frac{t}{D}}\right) \tag{S34}$$

which coincides with Eqn. (13) of the manuscript. In Eqn. (S34), $k_{\mathrm{T}} = k_{\mathrm{ox}} + k_{\mathrm{red}}$.

a.2. Thin-Layer (TL) limit for bounded conditions

By imposing the condition $p \to 0$ into equations (S31)-(S32) for the bounded case, and taking into account that $f^{\mathrm{B}}(p \to 0) = 1/\sqrt{p}$ [S8], it is obtained,

$$\frac{\bar{I}^{\mathrm{B,TL}}}{FA}\frac{L}{D}=\left(\tilde{k}_{\mathrm{ox}}c_{\mathrm{R}}^{*}-\tilde{k}_{\mathrm{red}}c_{\mathrm{O}}^{*}\right)\frac{1}{p+\tilde{k}_{\mathrm{T}}} \tag{S35}$$

which becomes, after the inverse of the Laplace transform, into:

$$I^{\mathrm{B,TL}}=Q_{\mathrm{F}}\left(\left(\frac{k_{\mathrm{ox}}}{L}\right)-\left(\frac{k_{\mathrm{red}}}{L}\right)\left(\frac{c_{\mathrm{O}}^{*}}{c_{\mathrm{R}}^{*}}\right)\right)e^{-\left(\frac{k_{\mathrm{T}}}{L}\right)t} \tag{S36}$$

with

$$Q_{\mathrm{F}} = FAc_{\mathrm{R}}^{*}L \tag{S37}$$

Eqn. (S36) is equivalent to Eqn. (17) in the manuscript.

By taking into account the expressions of rate constants k_{ox} and k_{red} given by Eqns. (S1) and (S5), it is possible to re-write Eqn. (S36) in the way,

$$I^{\mathrm{B,TL}} = Q_{\mathrm{F}}\left(\frac{k^{0'}}{L}\right)g(\eta)\left(e^{\eta}-\left(\frac{c_{\mathrm{O}}^{*}}{c_{\mathrm{R}}^{*}}\right)\right)e^{-\left(\frac{k^{0'}}{L}\right)g(\eta)\left(1+e^{\eta}\right)t} \tag{S38}$$

The operating rate constant in this limiting case is given by the ratio $\left(k^{0'}/L\right)$ with dimensions of inverse of time, such that the current depends on a dimensionless rate constant equal to $\bar{k}_{\mathrm{B,TL}}^{0'} = \left(k^{0'}/L\right)t$. This behavior is analogous to that observed for electroactive monolayers formed by the adsorption of a redox species R/O, a case for which the surface rate constant has dimensions of inverse of time [S3, S9].

a.3. TL limit for unbounded conditions

By imposing the condition $p \to 0$ into equations (S31)-(S32) for the unbounded case, and taking into account that $f^{\mathrm{U}}(p \to 0) = \sqrt{p}$ [S8], it is obtained,

$$\frac{\bar{I}^{\mathrm{U,TL}}}{FA}\frac{L}{D} = \frac{\tilde{k}_{\mathrm{ox}}c_{\mathrm{R}}^{*} - \tilde{k}_{\mathrm{red}}c_{\mathrm{O}}^{*}}{1+\tilde{k}_{\mathrm{T}}}\frac{1}{p} \tag{S39}$$

By carrying out the inverse of the Laplace transform is finally obtained,

$$I^{\mathrm{U,TL}} = FA\frac{D}{L}\frac{\tilde{k}_{\mathrm{ox}}c_{\mathrm{R}}^{*} - \tilde{k}_{\mathrm{red}}c_{\mathrm{O}}^{*}}{1+\tilde{k}_{\mathrm{T}}} \tag{S40}$$

which coincides with Eqn. (19) in the manuscript.

As in the previous limit case, by taking inserting the expressions of rate constants k_{ox} and k_{red} given by Eqns. (S1) and (S5) into Eqn. (S40) it is obtained,

$$I^{\mathrm{U,TL}} = FAc_{\mathrm{R}}^{*}\frac{D}{L}\frac{\left(\frac{k^{0'}L}{D}\right)g(\eta)\left(e^{\eta}-\frac{c_{\mathrm{O}}^{*}}{c_{\mathrm{R}}^{*}}\right)}{1+\left(\frac{k^{0'}L}{D}\right)g(\eta)\left(1+e^{\eta}\right)} \tag{S41}$$

In this case, the stationary current depends on a dimensionless rate constant equal to $\bar{k}_{\mathrm{U,TL}}^{0'} = k^{0'}\left(L/D\right)$.

b. Numerical calculation of the current.

Eqn. (S31) can be re-written in the way,

$$\bar{Y}^{\mathrm{B/U}} + \tilde{k}_{\mathrm{T}} \frac{f^{\mathrm{B/U}}(p)}{\sqrt{p}} \bar{Y}^{\mathrm{B/U}} = \frac{1}{p} \tag{S42}$$

where

$$\bar{Y}^{\mathrm{B/U}} = \frac{\bar{I}^{\mathrm{B/U}}}{FA} \frac{L}{D} \frac{1}{\tilde{k}_{\mathrm{ox}} c_{\mathrm{R}}^{*} - \tilde{k}_{\mathrm{red}} c_{\mathrm{O}}^{*}} \tag{S43}$$

By applying the inverse Laplace transform to Eqn. (S42) and applying the convolution theorem [S1, S8, S10], it is obtained,

$$\frac{1 - Y^{\mathrm{B/U}}}{\tilde{k}_{\mathrm{T}}} = \int_0^t \left(Y^{\mathrm{B/U}}(u) L^{-1} \left(\frac{f^{\mathrm{B/U}}(p)}{\sqrt{p}} \right)_{t-u} \right) du \tag{S44}$$

which becomes into (see Eqn. (S43)),

$$I^{\mathrm{B/U}} = FA \frac{D}{L} \left(\tilde{k}_{\mathrm{ox}} c_{\mathrm{R}}^{*} - \tilde{k}_{\mathrm{red}} c_{\mathrm{O}}^{*} \right) - \tilde{k}_{\mathrm{T}} \frac{D}{L} \int_0^t \left(I^{\mathrm{B/U}}(u) L^{-1} \left(\frac{f^{\mathrm{B/U}}(p)}{\sqrt{p}} \right)_{t-u} \right) du \tag{S45}$$

The operation $L^{-1}\left(f^{\mathrm{B/U}}(p) / \sqrt{p} \right)$ lead to the following expression (see Eqn. (S32) and [S8]),

$$L^{-1} \left(\frac{f^{\mathrm{B/U}}(p)}{\sqrt{p}} \right)_{t-u} = \frac{1}{\sqrt{\pi D (t-u)}} \left(1 + 2 \sum_{m=1}^{\infty} \Delta_m^{\mathrm{B/U}} e^{-m^2 L^2 / D(t-u)} \right) \tag{S46}$$

where

$$\begin{aligned} \Delta_m^{\mathrm{B}} &= 1 \\ \Delta_m^{\mathrm{U}} &= (-1)^m \end{aligned} \tag{S47}$$

By inserting Eqns. (S46)-(S47) into Eqn. (S45) it is obtained,

$$I^{\mathrm{B/U}} = FA \frac{D}{L} \left(\tilde{k}_{\mathrm{ox}} c_{\mathrm{R}}^{*} - \tilde{k}_{\mathrm{red}} c_{\mathrm{O}}^{*} \right) - \tilde{k}_{\mathrm{T}} \sqrt{\frac{D}{\pi}} \frac{1}{L} \int_0^t \left(I^{\mathrm{B/U}}(u) \, \mathrm{kernel}^{\mathrm{B/U}}(t-u) \right) du \tag{S48}$$

being

$$\mathrm{kernel}^{\mathrm{B/U}}(t-u) = \frac{1}{\sqrt{t-u}} \left(1 + 2 \sum_{m=1}^{\infty} \Delta_m^{\mathrm{B/U}} e^{-m^2 L^2 / D(t-u)} \right) \tag{S49}$$

In line with previous references, it is possible to re-write the expression of function $\mathrm{kernel}^{\mathrm{B/U}}(t-u)$ in a more compact way [S11, S12],

$$\mathrm{kernel}^{\mathrm{B}}\left(t-u\right)=\begin{cases}\dfrac{1}{\sqrt{t-u}} & \text{if } D\left(t-u\right)/L^{2}\leq 0.12\\ 1+2\left(e^{-\frac{\pi^{2}D(t-u)}{L^{2}}}+e^{-\frac{4\pi^{2}D(t-u)}{L^{2}}}\right) & \text{otherwise}\end{cases} \tag{S50}$$

$$\mathrm{kernel}^{\mathrm{U}}\left(t-u\right)=\begin{cases}\dfrac{1}{\sqrt{t-u}} & \text{if } D\left(t-u\right)/L^{2}\leq 0.12\\ 2\left(e^{-\frac{\pi^{2}D(t-u)}{4L^{2}}}+e^{-\frac{9\pi^{2}D(t-u)}{4L^{2}}}\right) & \text{otherwise}\end{cases} \tag{S51}$$

Eqn. (S48) can be solved in a numerical way by applying the Olmstead and Nicholson method [S13]. Thus, the total time is divided into n intervals of the same duration (τ), and the integral is converted into a sum of n sub-integrals, for each of which the current is assumed to be constant. After a small re-arrangement, the final expression is the following,

$$I_{n}^{\mathrm{B/U}}=\frac{I\left(0\right)-\tilde{k}_{\mathrm{T}}\sqrt{\dfrac{D}{\pi}}\dfrac{1}{L}\sum_{j=1}^{n-1}\left(I_{j}\mathrm{ikernel}^{\mathrm{B/U}}\left(t_{n,j}\right)\right)}{1+\tilde{k}_{\mathrm{T}}\sqrt{\dfrac{D}{\pi}}\dfrac{1}{L}\mathrm{ikernel}^{\mathrm{B/U}}\left(t_{n,n}\right)} \tag{S52}$$

where,

$$\begin{aligned}t_{n,j}&=\left(n-j\right)\Delta\tau\\ t_{n,n}&=\Delta\tau\end{aligned} \tag{S53}$$

and $\mathrm{ikernel}^{\mathrm{B/U}}$ being the integrated expressions of $\mathrm{kernel}^{\mathrm{B/U}}$ given by Eqns. (S50)-(S51),

$$\mathrm{ikernel}^{\mathrm{B}}\left(z\right)=\begin{cases}2\sqrt{\tau}\left(\sqrt{z+1}-\sqrt{z}\right) & \text{if } \left(Dz\Delta\tau/L^{2}\right)\leq 0.12\\ \Delta\tau+\\ \quad+\dfrac{2L^{2}}{\pi^{2}D}\left(e^{-\frac{\pi^{2}D}{L^{2}}z\Delta\tau}-e^{-\frac{\pi^{2}D}{L^{2}}(z+1)\Delta\tau}+\dfrac{1}{4}\left(e^{-\frac{4\pi^{2}D}{L^{2}}z\Delta\tau}-e^{-\frac{4\pi^{2}D}{L^{2}}(z+1)\Delta\tau}\right)\right) & \\ & \text{otherwise}\end{cases} \tag{S54}$$

$$\mathrm{ikernel}^{\mathrm{U}}\left(z\right)=\begin{cases}2\sqrt{\tau}\left(\sqrt{z+1}-\sqrt{z}\right) & \text{if } \left(Dz\Delta\tau/L^{2}\right)\leq 0.12\\ \dfrac{8L}{\pi^{2}D}\left(e^{-\frac{\pi^{2}D}{4L^{2}}z\Delta\tau}-e^{-\frac{\pi^{2}D}{4L^{2}}(z+1)\Delta\tau}+\dfrac{1}{9}\left(e^{-\frac{9\pi^{2}D}{4L^{2}}z\Delta\tau}-e^{-\frac{9\pi^{2}D}{4L^{2}}(z+1)\Delta\tau}\right)\right) & \\ & \text{otherwise}\end{cases} \tag{S55}$$

S2. Additional theoretical results

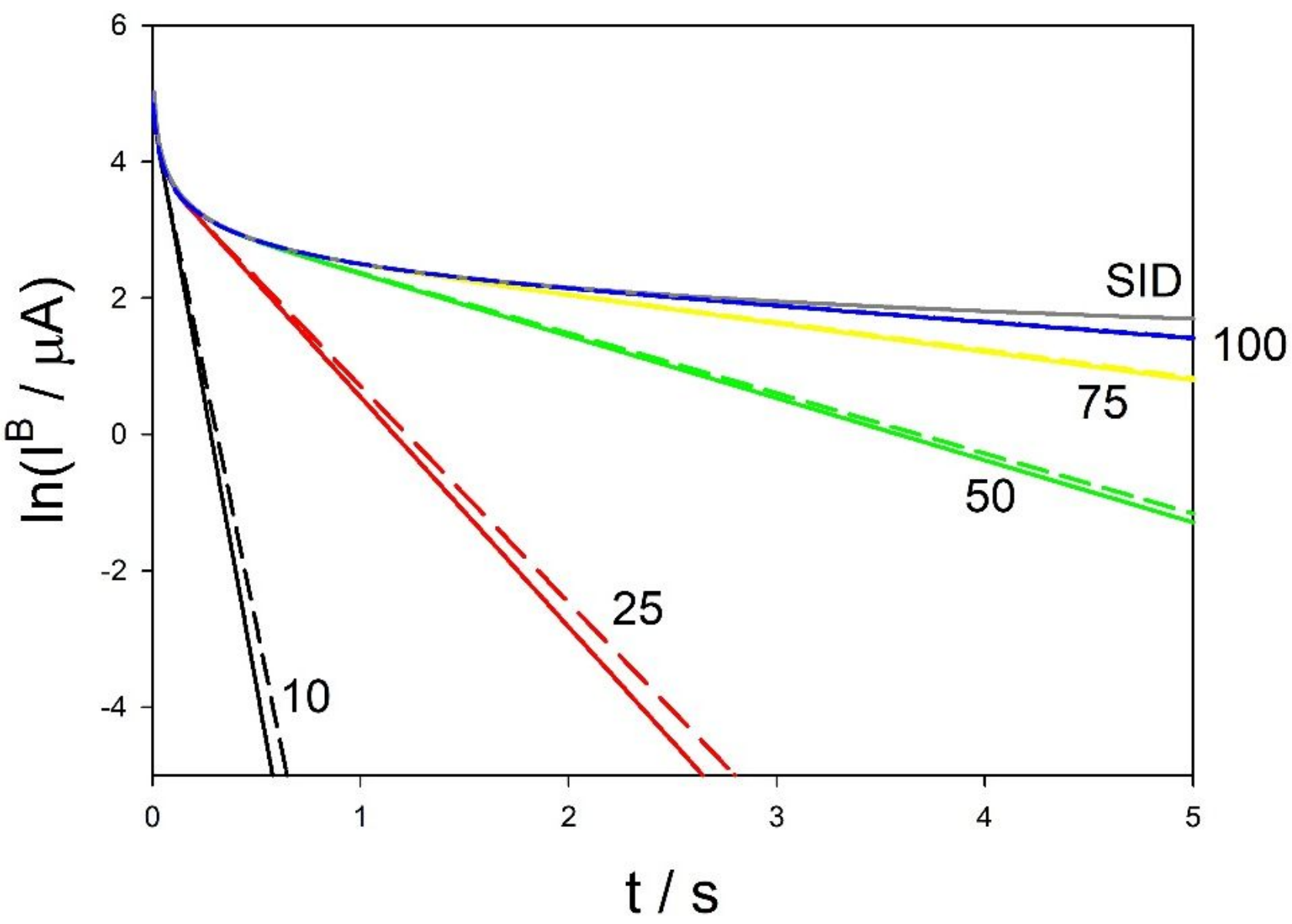

Figure S2. Influence of L for a fixed $k^{0'}$ value in the $\ln(I) - t$ curves for bounded mass transport mode calculated by using Eqns. (7)-(12) in the manuscript for BV (solid lines) and MHC (dashed lines) formalisms. $k^{0'} = 10^{-3}$ cm s^{-1}, α=0.5, λ=1 eV, γ=0, $D = 10^{-5}$ cm^2 s^{-1}, $\Delta E = E - E^{0'} = 0.200$ V, $c^* = 1.0$ mM, A=0.0707 cm^2, T=298 K. The values of L (in microns) are given in the Figure. The curves corresponding to SID conditions (grey lines) have been included for comparison.

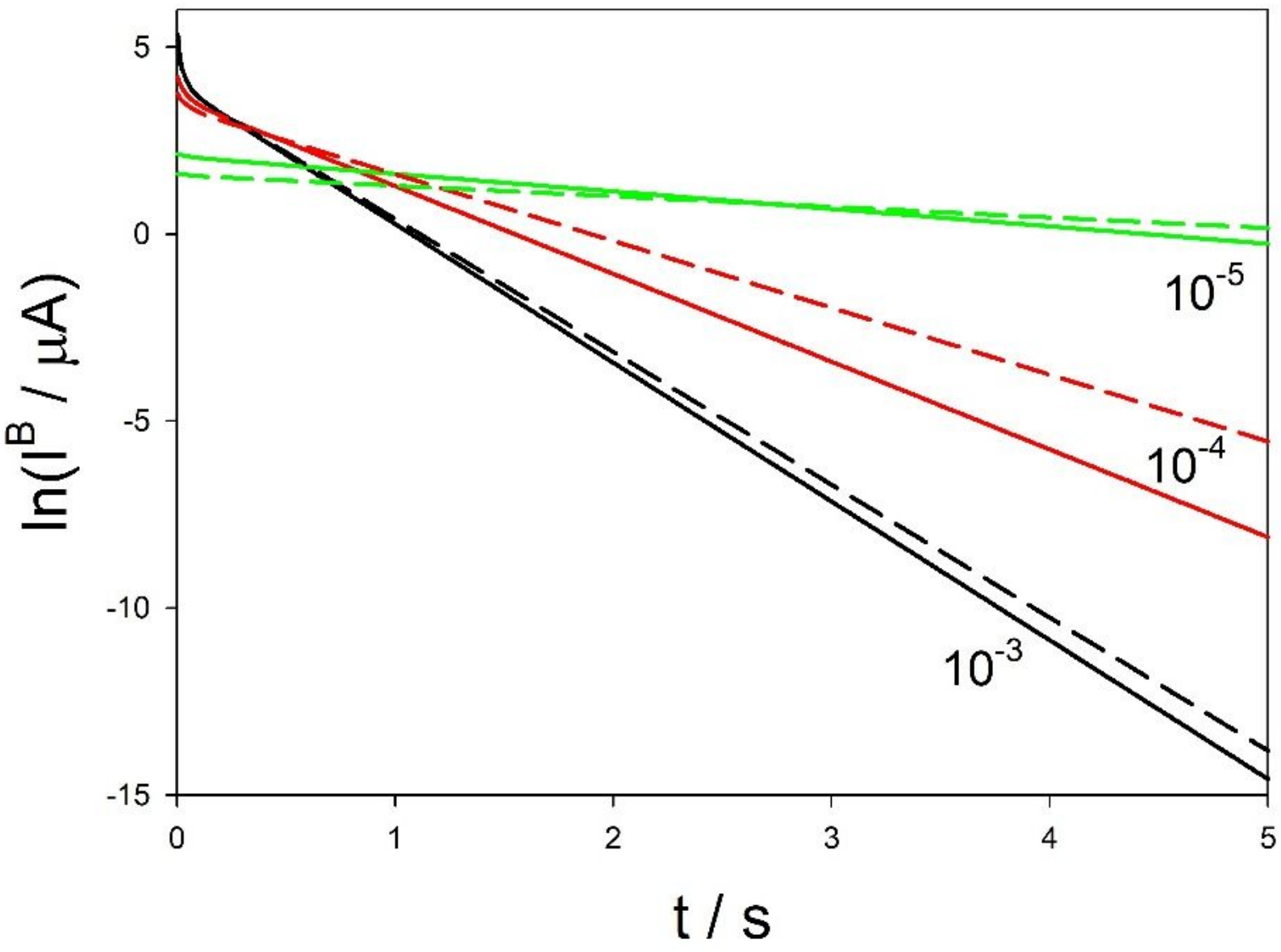

Figure S3. Influence of $k^{0\prime}$ for a fixed value of L in the $\ln\left(I\right)-t$ curves for bounded mass transport mode calculated by using Eqns. (7)-(12) in the manuscript for BV (solid lines) and MHC (dashed lines) formalisms. $L=25$ μm, α=0.5, λ=1 eV, γ=0, $D=10^{-5}$ cm^2 s^{-1}, $\Delta E = E - E^{0\prime} = 0.250$ V, $c_R^* = 1.0$ mM, A=0.0707 cm^2, T=298 K. The values of $k^{0\prime}$ (in cm s^{-1}) are given in the Figure.

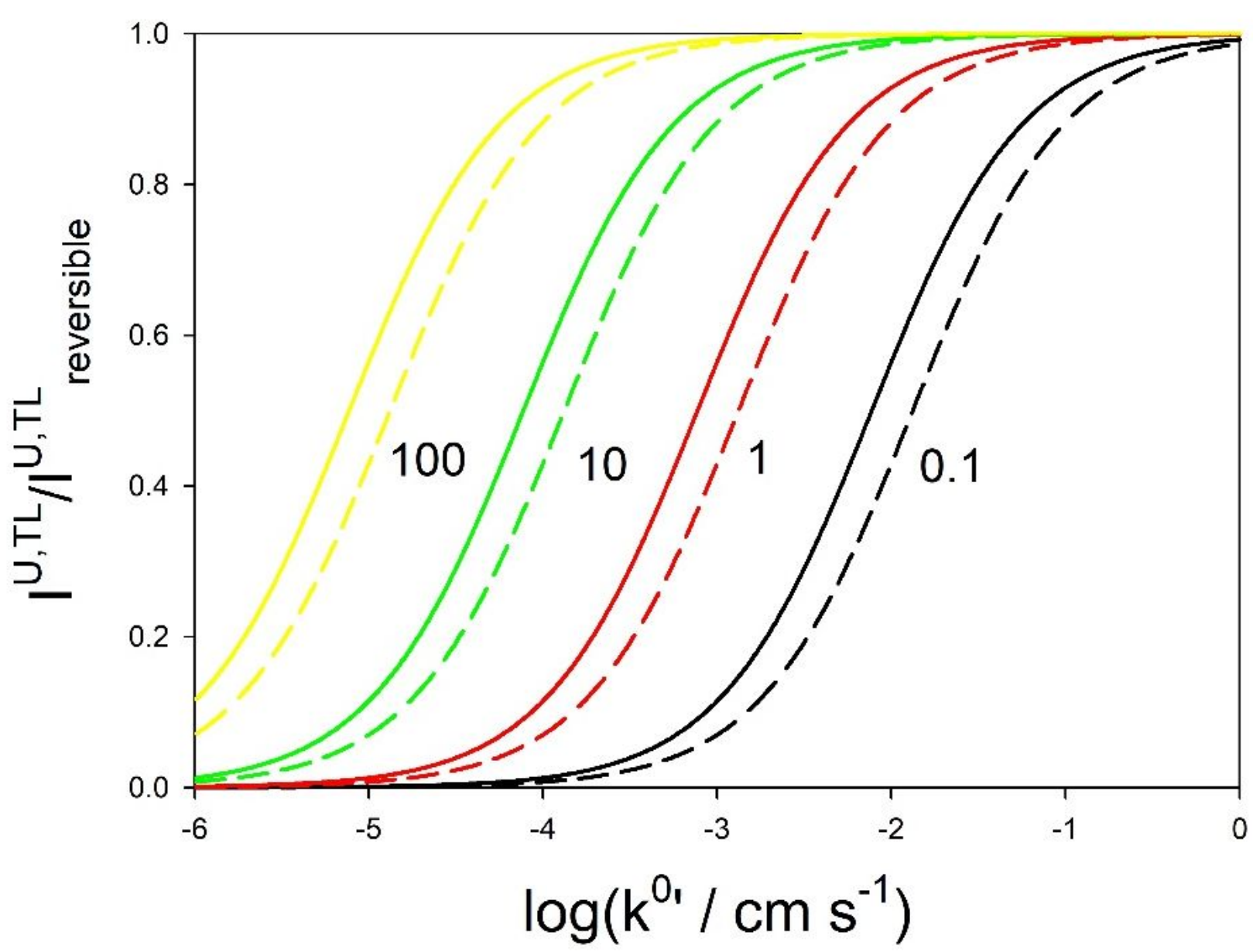

Figure S4. Evolution of the stationary current obtained under unbounded conditions with $\log\left(k^{0\prime}\right)$, calculated from Eqn. (20) in the manuscript for a symmetrical BV (solid lines) and MHC (dashed lines) formalisms for different values of *L* in microns (values shown in the Figure). $\Delta E = E - E^{0\prime} = 0.250$ V, $c_{\mathrm{O}}^{*} = 0$, α=0.5, λ=1 eV, γ=0, $D = 10^{-5}$ cm^2 s^{-1}.

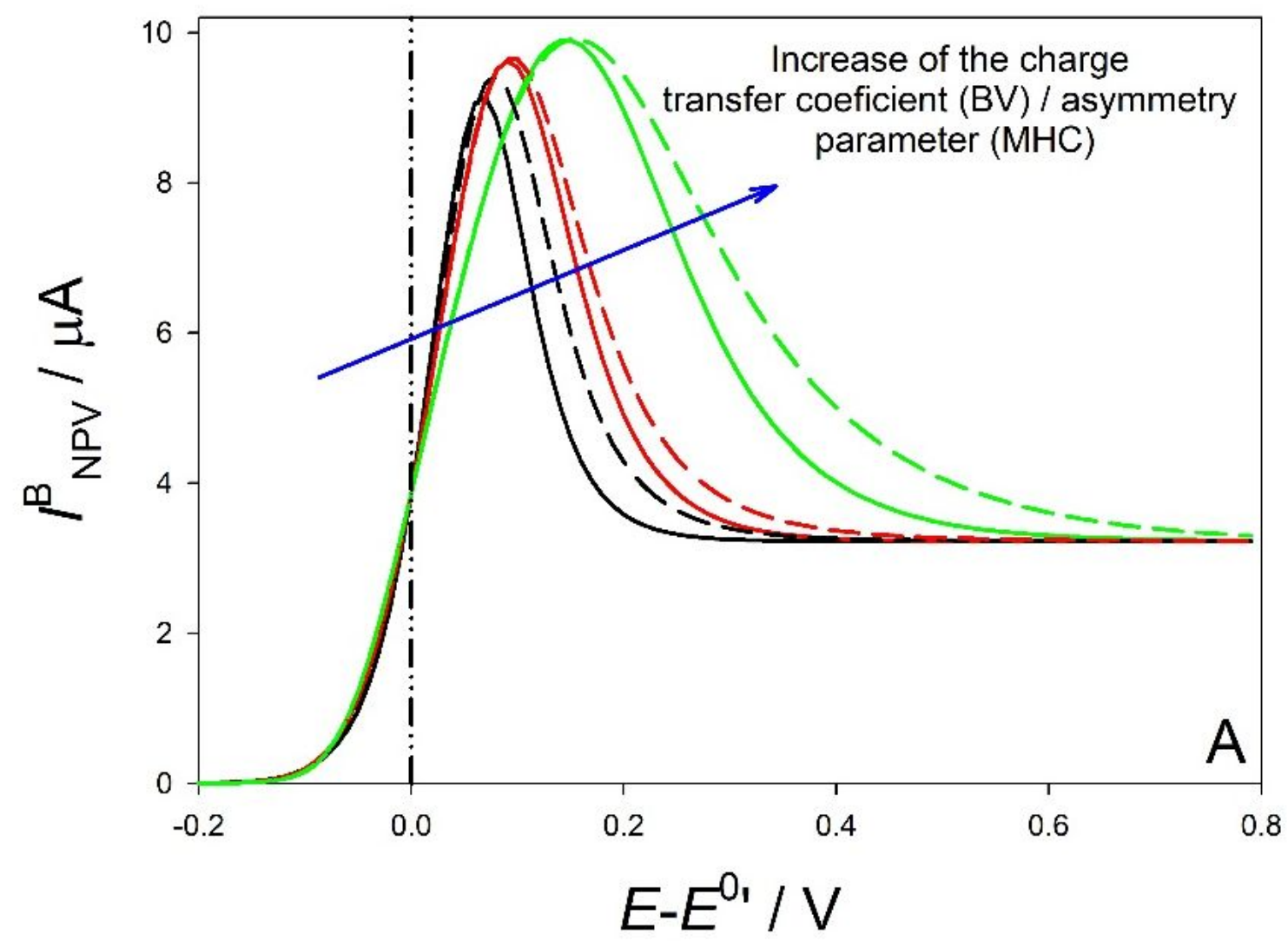

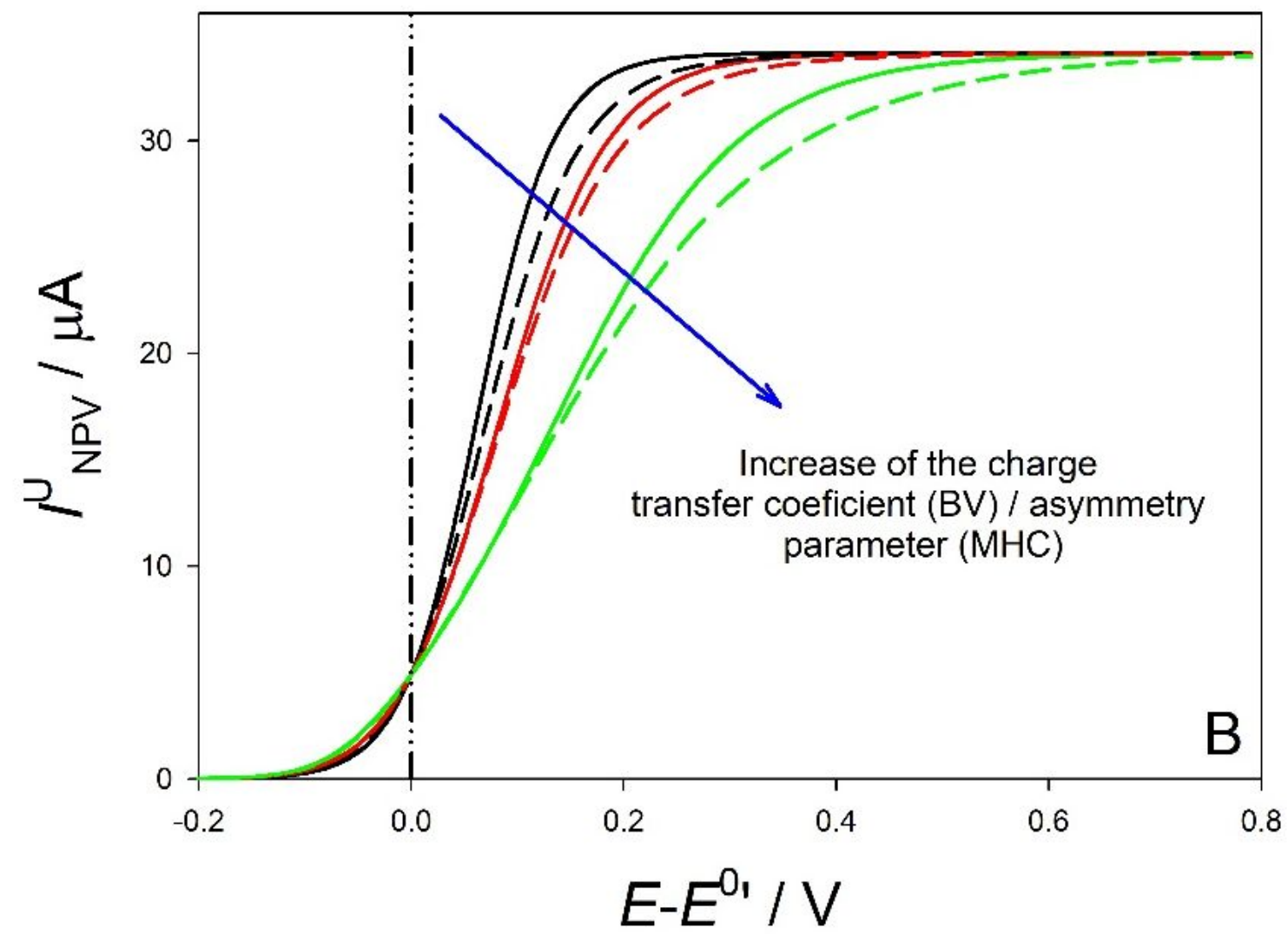

Figure S5. Influence of the symmetry of the charge transfer in the NPV curves for bounded (A) and unbounded (B) mass transport modes calculated by using Eqns. (7)-(13) in the manuscript for BV (solid lines) and MHC (dashed lines) formalisms. $L = 25$ µm, $k^{0\prime} = 10^{-3}$ cm s^{-1}, λ=1 eV, $D = 10^{-5}$ cm^2 s^{-1}, $\Delta E = 10$ mV, $\tau = 0.5$ s, $c_R^* = 1.0$ mM, A=0.0707 cm^2. The values of the charge transfer coefficient α (BV) / asymmetry parameter γ (MHC) are: black lines, 0.3 / -0.5; red lines, 0.5 / 0; green lines, 0.7 / +0.85. Vertical black dashed-dotted line marks the location of the formal potential.

S4. Experimental conditions

Reagents and Chemicals

4-Carboxy-2,2,6,6-tetramethylpiperidine 1-oxyl (radical 4-Carboxy-TEMPO) and KCl (Sigma-Aldrich) were reagent grade and used as received.

Electrochemical measurements and data analysis

Normal Pulse Voltammetry, Cyclic Voltammetry and Chronoamperometry were performed by using a home-made computer-driven potentiostat-galvanostat (Scientific Instrumentation Service of University of Murcia). All experiments were performed in presence of oxygen. A three-electrode set-up were employed in the experiments with a 3 mm Boron-Doped Diamond electrode (BDD, Windsor Scientific) acting as the working electrode. The counter electrode was a Pt wire, and the reference electrode was an Ag/AgCl (KCl sat.). The electrochemical FD cell used for the bounded configuration is a homemade PVC single-compartment cell with a total volume of 2 mL (see Fig. S6). The FD cell for the unbounded configuration is a circular cell in which a gold electrode (diameter=3.0 mm) has been inserted at the bottom, located below the BDD working electrode. This gold electrode is controlled potentiostatically (Fig. S7). The piezoelectric system used to perform FD experiments comes from Scanning Electrochemical Workstation, Model 370, Princeton Applied Research. The working electrode was mechanically polished on alumina slurry (1.0 and 0.05 µm, Buehler), washed with water and cleaned electrochemically by cycling the potential between 0 V and -1.0 V in 1.0 M NaOH solution until a stable voltammogram was obtained.

The NPV potential waveform consists in the application of several constant potentials (of increasing amplitude) with identical time length τ. Between the application of two potentials of this sequence, the electrode is kept at a rest potential at which the regeneration of the faradaic converted species takes place during a time t_{rest} (see Fig. S8). This time interval between two consecutive potentials at which E_{rest} is applied should be much greater than τ under SID conditions, for the initial conditions to be restored. Since the charge transfer processes is analysed under FD conditions, the regeneration of the redox species is complete in a relatively short time (2.0 s for the experimental conditions followed here), such that each individual potential of the complete sequence is equivalent to a single experiment with bulk concentrations of the species redox.

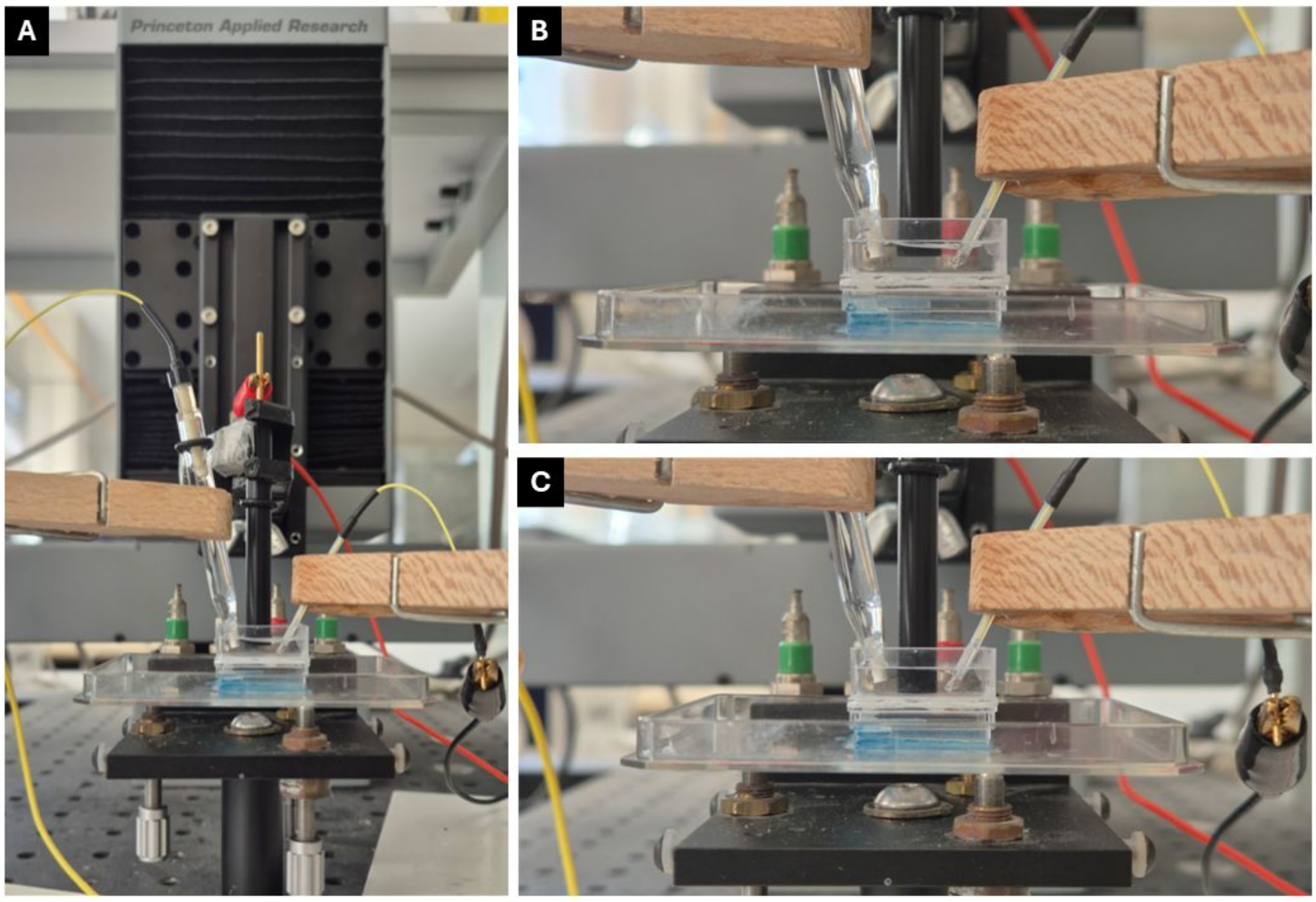

Figure S6. Photograph of the experimental setup for a bounded configuration. Working electrode: BDD electrode (GC); reference electrode: Ag/AgCl (saturated KCl); counter electrode: Pt wire. (A) General view of the experimental system. (B) Detailed view of the electrochemical cell under the semi-infinite diffusion (SID) configuration. (C) Detailed view of the electrochemical cell under FD configuration.

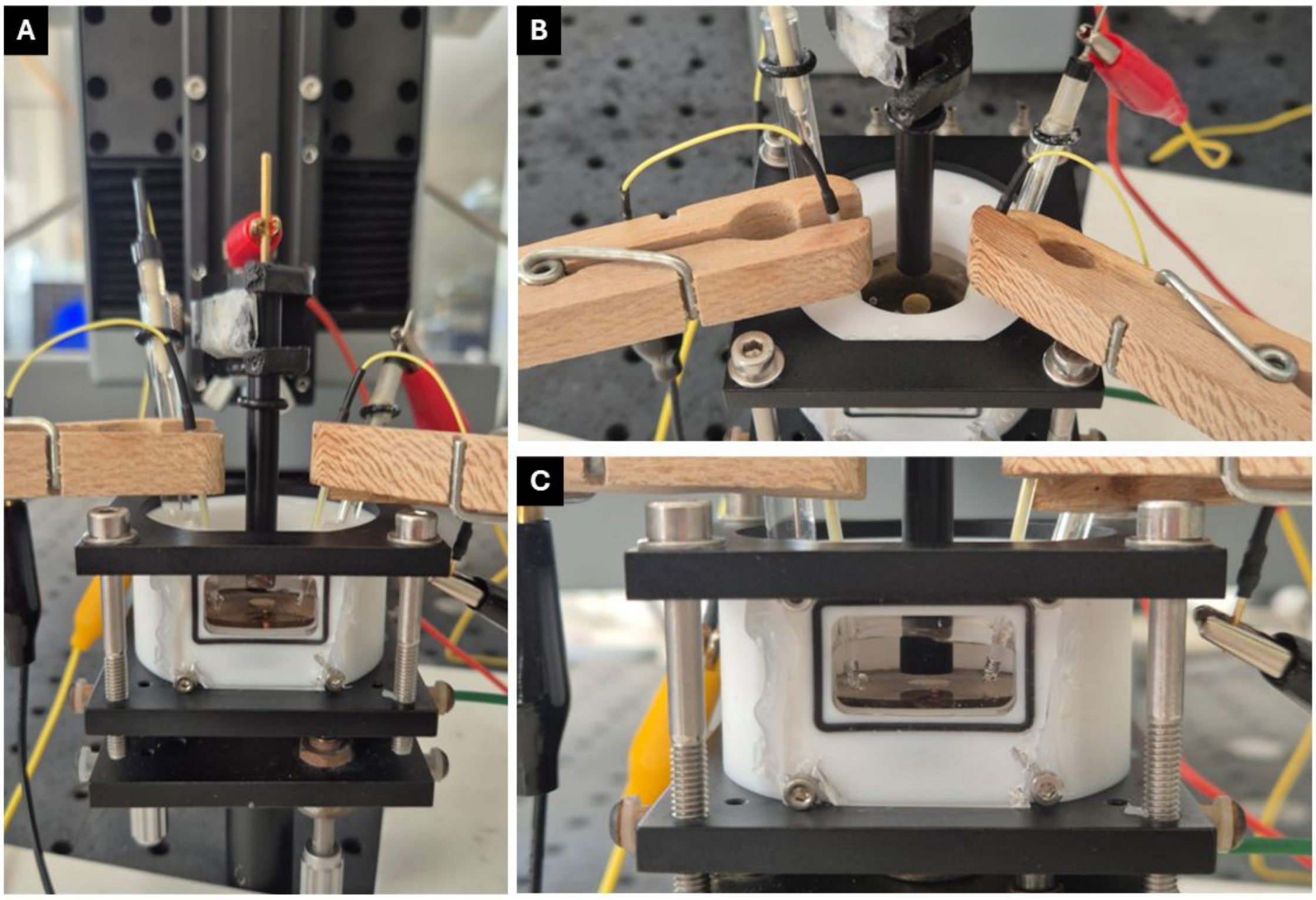

Figure S7: Photograph of the experimental setup in the unbounded configuration. Upper working electrode: BDD (GC); lower working electrode: gold disc (Au); reference electrodes: Ag/AgCl (saturated KCl); counter electrodes: Pt wire. (A) General view of the experimental system. (B) Detailed view of the electrochemical cell assembly. (C) Lateral view of the cell showing the facing electrode configuration.

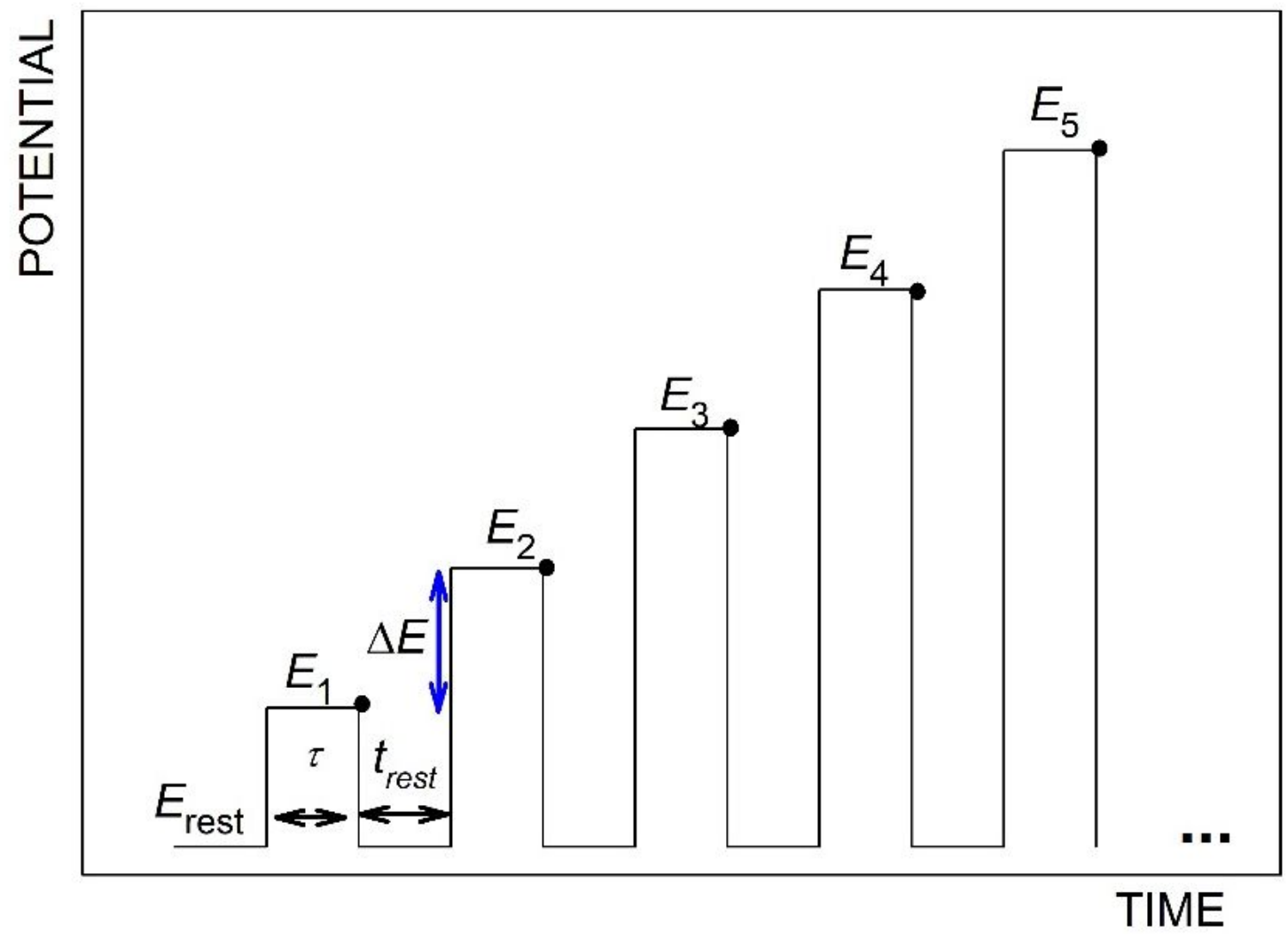

Figure S8. Scheme of the potential-time waveform for Normal Pulse Voltammetry in its multipulse modality [S3]. Each E_i potential (i=1, 2, …) is applied during the same time, τ. $\Delta E = E_{i+1} - E_i$ is a constant value. E_{rest} is a potential applied during a time t_{rest} at which the regeneration of the faradaic converted species takes place at the working electrode surface. Black dots indicate the times at which the current is sampled.

S5. Chronoamperometric analysis for bounded conditions

The analysis of chronoamperometric responses under FD conditions present some problems derived from the influence of uncompensated resistance on the response, mainly at short times for which the current is high.

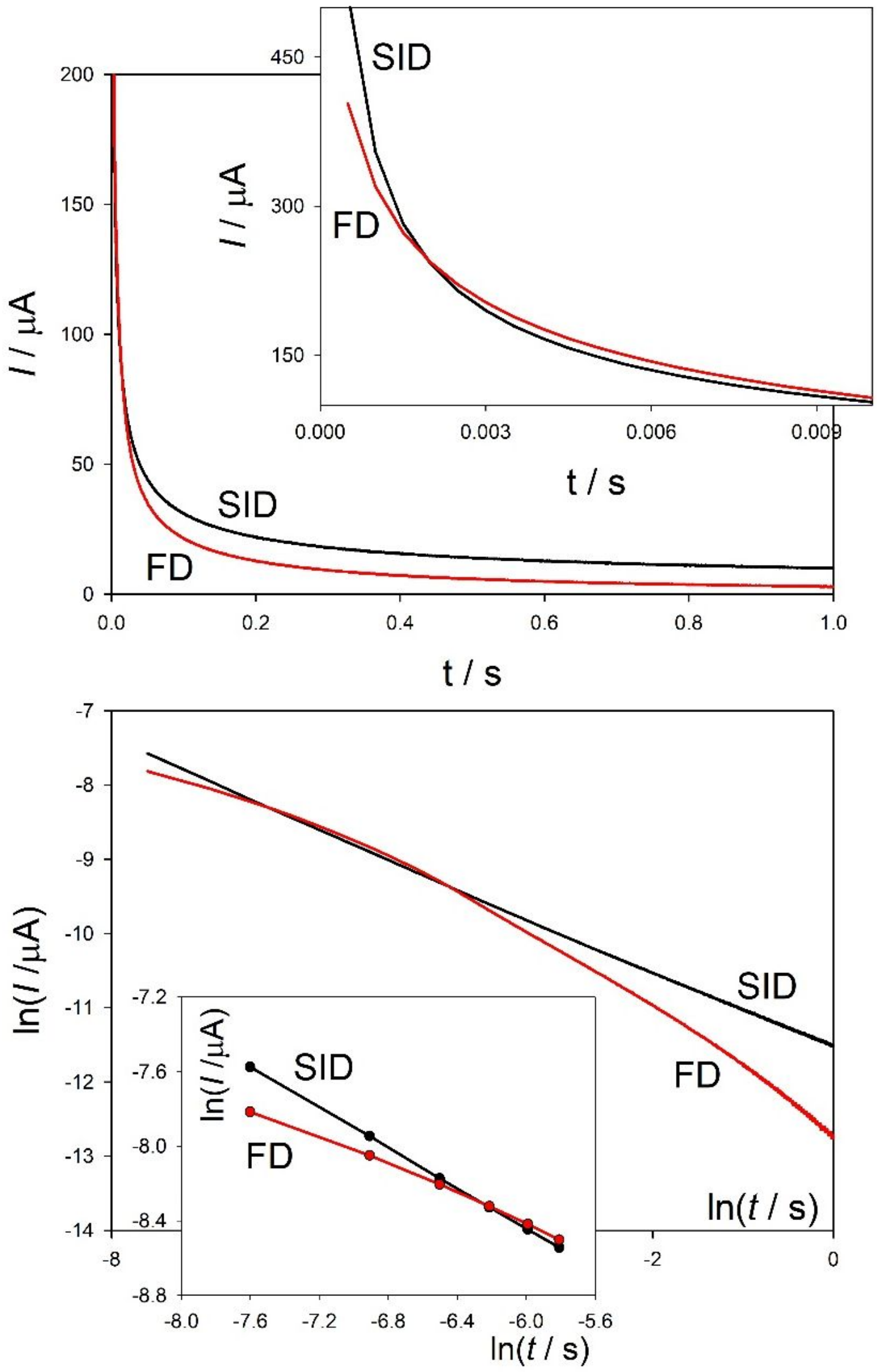

Figure S9. Chronoamperometric $I-t$ (upper Fig.) and $\ln(I)-\ln(t)$ (lower Fig.) curves corresponding to the application of a potential $E=1.100$ V (vs. Ag/AgCl, KCl (sat)) to a BDD electrode (3 mm diameter) in a solution with 4CT 1.07 mM + KCl 1.0 M under SID (black lines) and FD (12 µm, red lines) conditions. Inner Figs. show the two responses for a time interval of 50 ms. T=298 K.

Figure S9 shows the $I-t$ (upper Fig) and $\ln(I)-\ln(t)$ (lower Fig.) responses of 4CT 1.07 mM for the application of a constant potential corresponding to limiting current conditions

(E=1.100 V). The cronoamperograms have been recorded under SID (black lines) and FD (red lines, L=12 μm) conditions. Inner Figs. show the responses obtained for the first 10 ms.

As can be seen, at short times the currents are very high (above 100 μA), and they do not coincide, with the FD response being smaller than the SID one for times up to 2.5 ms. These differences are related to the RC component of the response, that dominates the current at short times. As previously reported[S14, S15], for small distances, there is an increase of the uncompensated resistance in the current leading to a smaller value of the current. This distortion can be also seen in Fig. S10, which compares the measured currents obtained under SID and FD conditions for different potentials for times below 50 ms.

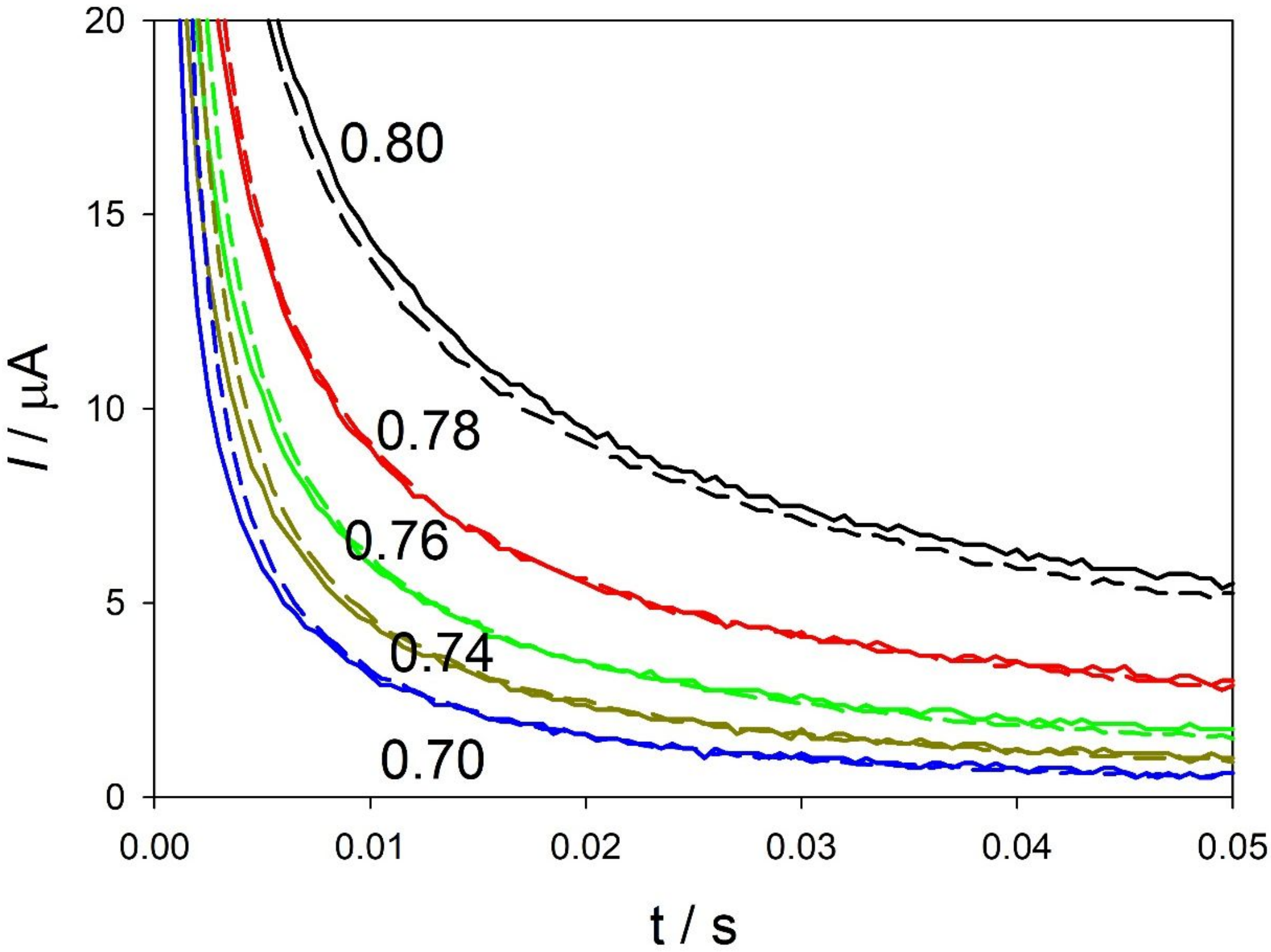

Figure S10. Chronoamperometric $I-t$ curves corresponding to the application of different potentials (values in V vs. Ag/AgCl, KCl (sat) indicated in the curves) to a BDD electrode (3 mm diameter) in a solution with 4CT 1.07 mM + KCl 1.0 M under SID (solid lines) and FD (12 μm, dashed lines) conditions. T=298 K.

The I-t curves in Fig. S10 only becomes coincident for FD and SID conditions for times above 20 ms and potentials that are far removed from the system's nominal potential, conditions under which the measured current drops sharply. Indeed, the influence of uncompensated resistance for the FD configuration becomes negligible for long enough measurement times such that the current was below 100 μA.

S6. Additional experimental results

Table S1 shows the peak parameters for the CV curves in Fig. S11. The values of the $\Psi_{\text{Nicholson}}$ parameters have been obtained from the measurement of the peak-to-peak separation of the voltammograms recorded for different scan rates [S16].

Table S1. Values of the peak potentials for the direct and reverse scans, of the peak current of the direct scan and of different combinations of potentials for the CV curves shown in Fig. S11. The formal potential has been obtained as the semi-sum of the peak potentials for the direct and reverse scans.

Scan rate / V s^{-1}	$E_{\text{peak,a}}$ /V	$I_{\text{peak,a}}$ / μA	$E_{\text{peak,c}}$ /V	ΔE_{peak} /V	$E^{0\prime}$ /V	$\Psi_{\text{Nicholson}}$	$k^{0\prime} \times 10^3$ /cm s^{-1}
0.050	0.938	12.14	0.848	0.0895	0.893	0.768	2.87
0.100	0.944	16.70	0.844	0.0995	0.894	0.565	2.99
0.150	0.949	20.04	0.840	0.1092	0.894	0.448	2.90
0.200	0.952	22.79	0.836	0.1160	0.894	0.386	2.89

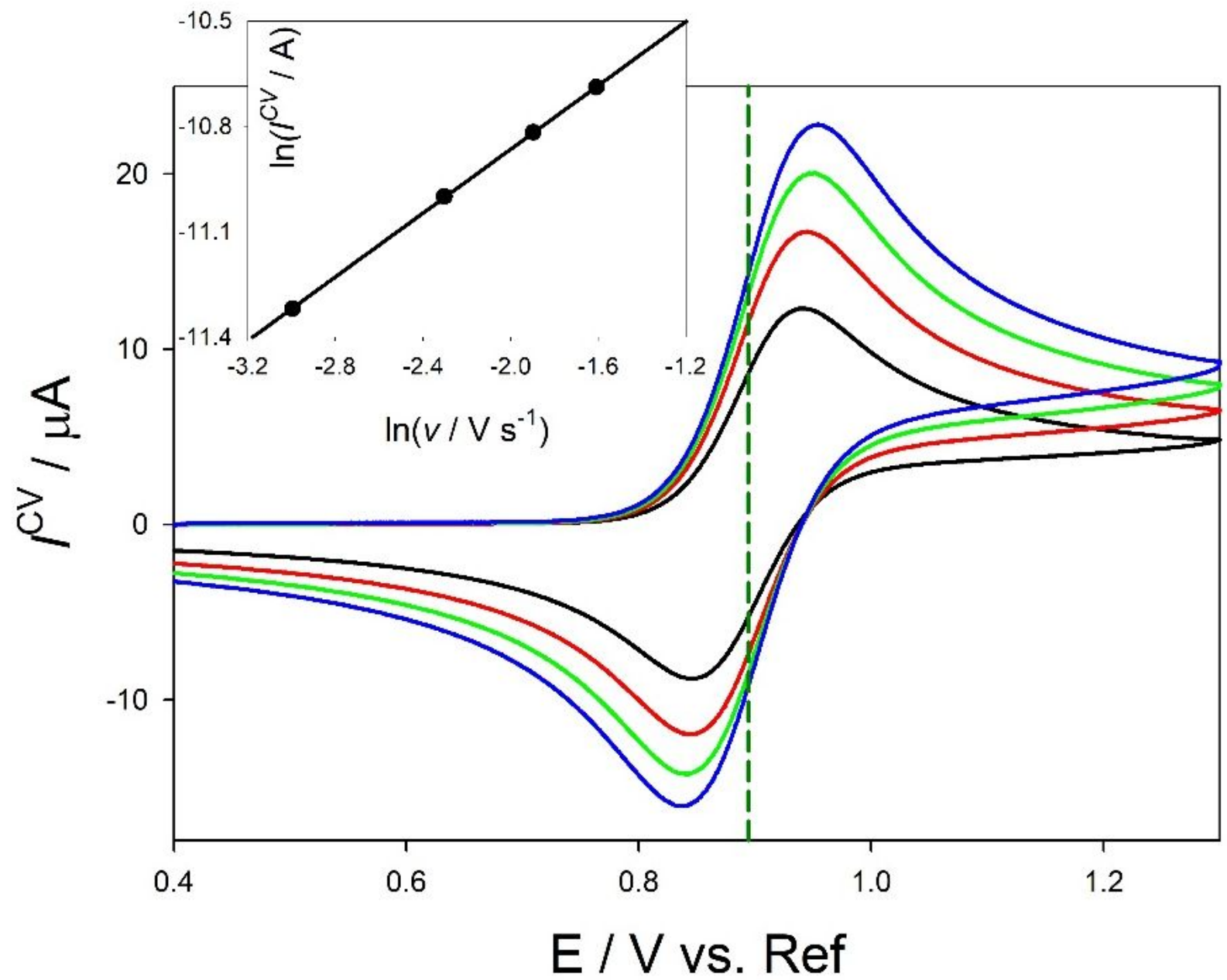

Figure S11. CV curves of 4CT 2.085 mM + KCL 1.0 at a boron doped electrode under SID conditions. The scan rates used (in mV s^{-1}) are: 50 (black line); 100 (red line); 150 (green line); 200 (blue line). Inner Fig.: $\ln\left(I_{\text{CV,peak}}^{\text{SID}}\right) - \ln\left(v\right)$. Vertical green dashed line marks the position of the formal potential.

The logarithmic Randles-Sevcik plot shown in the inner Fig. S10 shows an excellent linearity with a slope of 0.45, very close to the value 0.5 typical of SID diffusion.

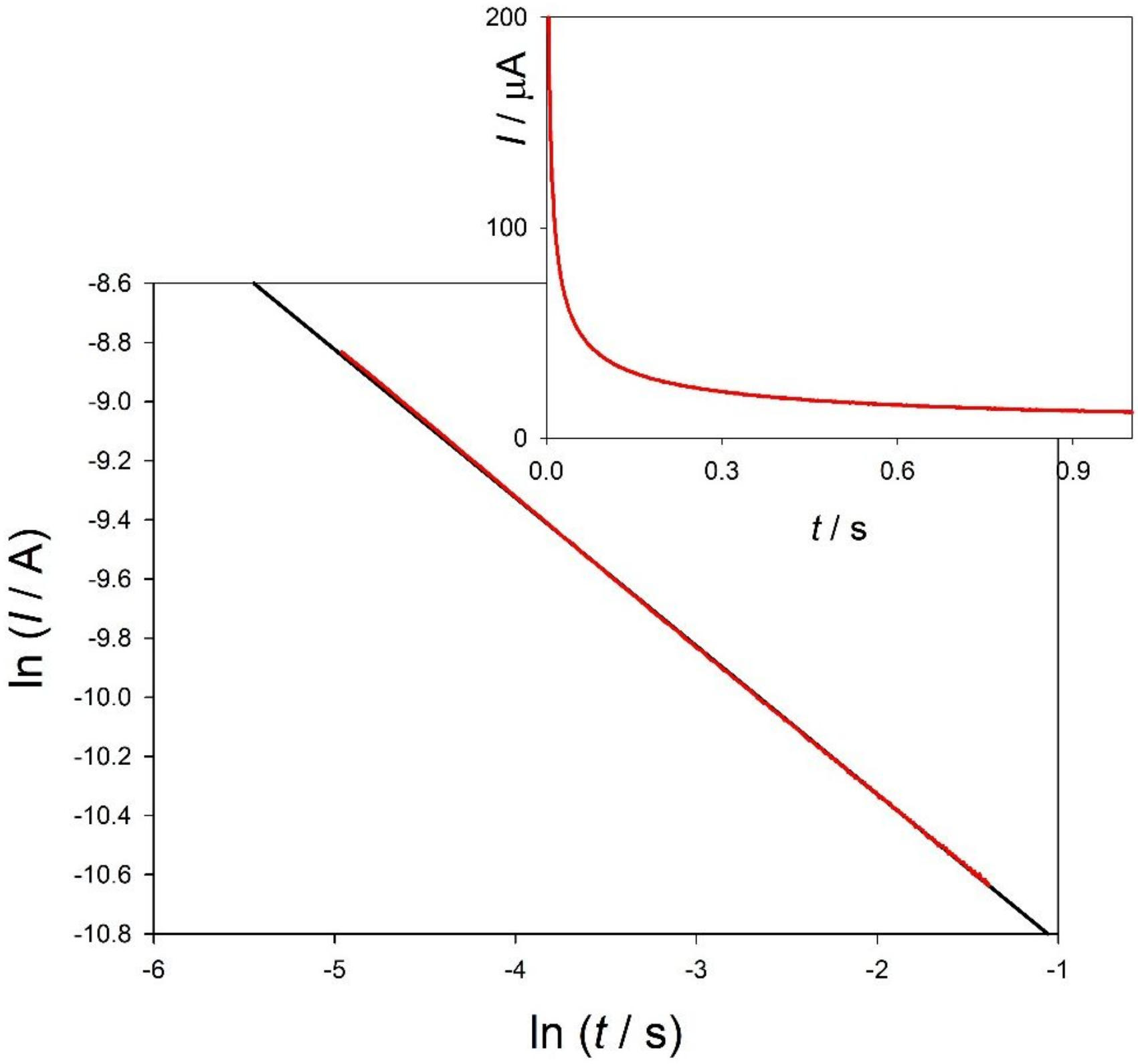

Figure S12. Chronoamperometric *I*-*t* (inner) and $\ln(I)-\ln(t)$ curve of 4CT 2.085 mM + KCl 1.0 at a Diamond Boron-Doped electrode under SID conditions. $E_{\mathrm{applied}}=1.3$ V. The slope of the logarithmic curve is -0.501, which perfectly agrees with the theoretical value for Cottrell equation.

S7. Fitting protocol

The protocol followed for the estimation of the different governing parameters of charge transfer processes is the following:

1. The diffusion coefficient has been previously obtained from the analysis of the current-time curves under SID conditions for the oxidation of 4CT by using the logarithmic form of the Cottrell's equation, see Fig. S12.

$$\ln(I) = \ln\left(FAc^{*}_{4\mathrm{CT}}\sqrt{\frac{D_{4\mathrm{CT}}}{\pi}} \right) - \frac{1}{2}\ln(t) \quad \mathbf{(S56)}$$

From the corresponding linear regression, the slopes of the corresponding plots show in all the cases, slopes close to the theoretical value -0.5 and the value of $D_{4\mathrm{CT}}$ has been obtained from the intercept.

2. The formal potential is initially estimated from the peak-to-peak difference observed in the CV curves for the oxidation of 4CT under SID conditions, see Fig. S11. This value is further refined in the fitting process. The value of the reorganization energy, λ, has been assumed equal to 1.0 eV, in line with typical values for organic molecules.

3. The operating value of the thickness of the solution in the FD assays is obtained from the bounded or unbounded limiting currents, see Figs. S13, region A, by using Eqns. (25) or (26) in the manuscript.

4. For the bounded case, the $I^{\mathrm{B}}_{\mathrm{NPV,peak}}$ value does not depend on the rate constant for values of $k^{0'} \leq 10^{-3}$ cm s^{-1}, see Figs. 5 and 6, and depends slightly on the symmetry degree of the process, see Fig. S5. Therefore, the value of $k^{0'}$ can be estimated from the location of the $E^{\mathrm{B}}_{\mathrm{NPV,peak}}$, which varies linearly with $\log\left(k^{0'}\right)$ (see Fig. 6) (region B).

5. In the unbounded case, the location of the half-wave potential, $E^{\mathrm{U}}_{\mathrm{NPV,half\,wave}}$ depends on the rate constant (region B). Therefore, the value of $k^{0'}$ can be estimated from values that give an adequate location of the theoretical curves.

6. The symmetry of the charge transfer (through the value of charge transfer coefficient α for a BV approach, or through the asymmetry parameter γ for a MHC one), affects the slope of the NPV curves in region C. Additionally, for the bounded case, it affects the value of the half-peak width of the peak observed. Once the location of the theoretical curve is adequate, the value of α / γ is adjusted in order to reproduce the half-peak width (bounded case) and the slope of the rising zone of the NPV response (bounded / unbounded cases).

7. This sequential process provide seed values of the rate constant, the formal potential and the symmetry parameters. These values are subsequently refined through a simple iterative fitting procedure applied to the full experimental voltammograms.

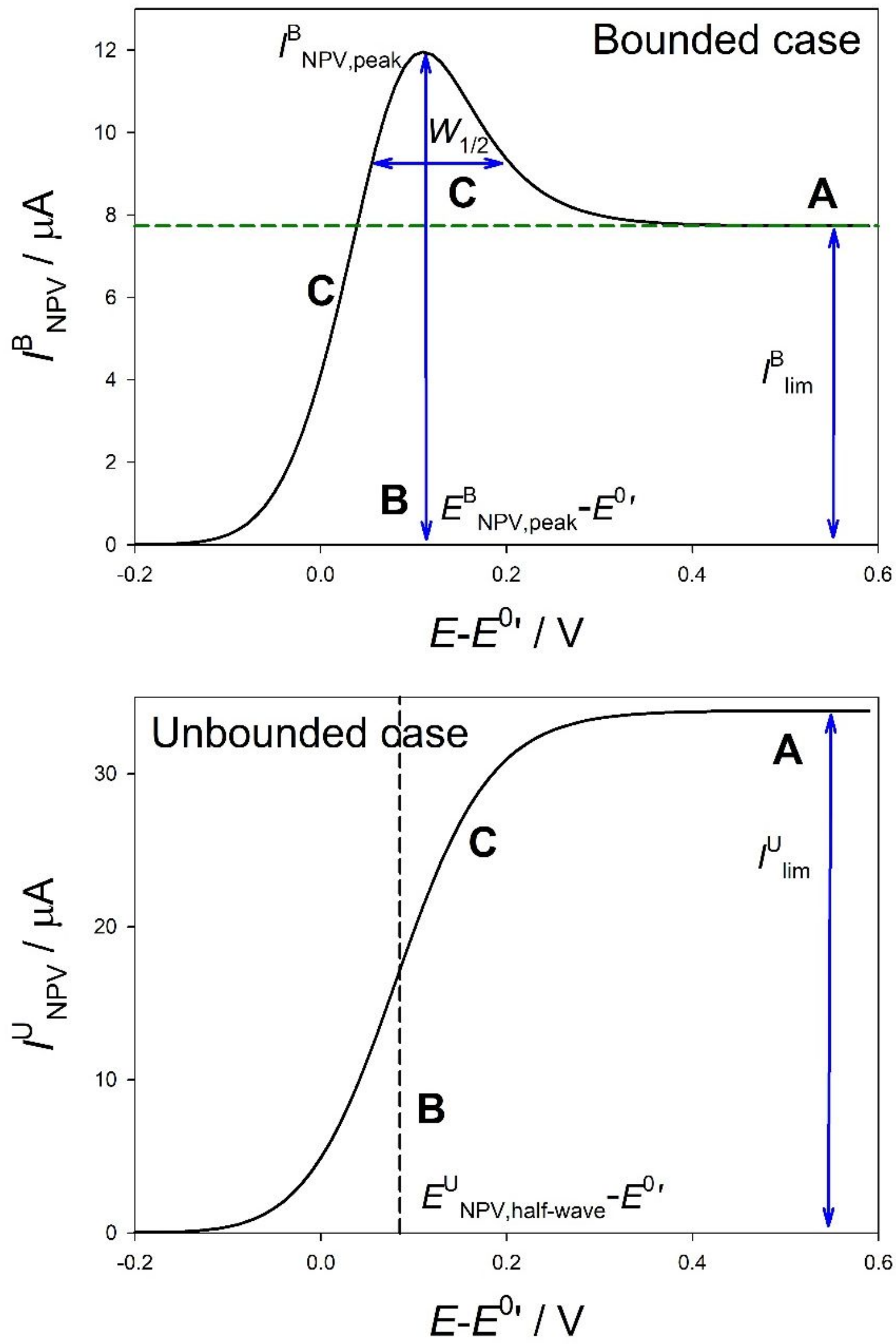

Figure S13. Characteristic features of a NPV curve for a quasi-reversible charge transfer process under bounded and unbounded conditions. Characteristic regions have been labelled (A, B and C) for the sequential fitting process. $k^{0\prime} = 10^{-3}$ cm s^{-1}, $\lambda = 1$ eV, $\gamma = 0$, $\tau = 0.5$ s, $A = 0.0707$ cm^2, $c^*_R = 1.0$ mM, $L = 10$ μm, $D = 10^{-5}$ cm^2 s^{-1}, T=298 K.

S8. References

S1. Bard, A. J.; Faulkner, L. R.; White, H. S. *Electrochemical Methods: Fundamental and Applications*, 3rd Ed, John Wiley & Sons, Inc., **2022**.

S2. Compton, R. G.; Banks, C. E. *Understanding Voltammetry*, 3rd Ed, World Scientific Publishing, **2018**.

S3. Molina, A.; Gonzalez J. *Pulse Voltammetry in Physical Electrochemistry and Electroanalysis*, Springer, **2016**.

S4. Laborda, E.; Henstridge, M.; Batchelor-McAuley, C.; Compton, R. G., Asymmetric Marcus-Hush theory for voltammetry, *Chem. Soc. Rev*. **2013**, *42* 4894-4905.

S5. Dickinson, E. J. F.; Wain, A. J. The Butler-Volmer equation in electrochemical theory: Origins, value, and practical application, *J. Electroanal. Chem*. **2020**, *872*, 114145.

S6. Oldham, K. B.; Myland, J. C. On the evaluation of the Marcus−Hush−Chidsey integral. *J. Electroanal. Chem*. **2011**, *655*, 65−72.

S7. Feldberg, S. W. Implications of Marcus−Hush theory for steady-state heterogeneous electron transfer at an inlaid disk electrode. *Anal. Chem*. **2010**, *82*, 5176−5183.

S8. Oldham, K. B.; Myland, J.; Spanier, J. An Atlas of functions, 2nd edition, **2000**, Springer, New York.

S9. Gonzalez, J.; Abenza, N.; Molina, A., Analytical solutions of the multipotential pulse quasi-reversible Q-E-t and I-E-t responses of strongly adsorpted redox molecules, *J. Electroanal. Chem*., **2006**, *596*, 74-86.

S10. Savéant, J. M.; Costentin, C. Elements of Molecular and Biomolecular Electrochemistry: An Electrochemical Approach to Electron Transfer Chemistry, 3rd ed, Wiley, **2019**.

S11. Hernandez-Tovar, J. V.; Martinez-Garcia, A. J.; Lopez-Tenes, M.; Martinez-Ortiz, F.; Molina, A.; Gonzalez, J. From Semi-Infinite to Thin-Layer Diffusion Effects of Finite Mass Transport on the Electrochemical Response of Redox Probes: Implications for Electroanalytical Measurements, *Anal. Chem*. **2025**, *97*, 2941–2951.

S12. Hernandez-Tovar, J. V.; Martinez-Garcia, A. J.; Lopez-Tenes, M.; Martinez-Ortiz, F.; Molina, A.; Gonzalez, J., Cyclic staircase voltammetry and cyclic voltammetry in current and charge modes for the analysis of the responses of fast redox probes under finite diffusion, *Electrochim. Acta* **2025**, *525*, 146157.

S13. Olmstead, M. L.; Nicholson, R. S. A method based on polynomial approximations for numerical solution of Volterra integral equations, *J. Electroanal. Chem. Interfacial Electrochem*., **1968**, *16*, 145-151.

S14. Yamada, K.; Kitamura, F.; Ohsaka, T.; Tokuda, K., Response Time of Thin-Layer Electrolytic Cells to Potential-Step Signals, *J. Electrochem. Soc*. **1996**, *143*, 4006-4012.

S15. Montella, C. Discussion of the potential step method for the determination of the diffusion coefficients of guest species in host materials: Part I. Influence of charge transfer kinetics and ohmic potential drop, *J. Electroanal. Chem*., **2002**, *518*, 61-83.
S16. Agarwal, R. The Nicholson Method of Determination of the Standard Rate Constant of a Quasireversible Redox Couple Employing Cyclic Voltammetry: Everything One Needs to Know!, *ACS Electrochem*. **2025**, *1*, 1885–1894.
